# New Features on the Environmental Regulation of Metabolism Revealed by Modeling the Cellular Proteomic Adaptations Induced by Light, Carbon, and Inorganic Nitrogen in *Chlamydomonas reinhardtii*

**DOI:** 10.3389/fpls.2016.01158

**Published:** 2016-08-09

**Authors:** Stéphanie Gérin, Pierre Leprince, Francis E. Sluse, Fabrice Franck, Grégory Mathy

**Affiliations:** ^1^Laboratory of Bioenergetics, Department of Life Sciences, Faculty of Sciences, University of LiegeLiege, Belgium; ^2^Laboratory of Nervous System Disorders and Therapy, Faculty of Medicine, GIGA-Neurosciences, University of LiegeLiege, Belgium; ^3^Upstream Process Sciences, UCB PharmaBraine l'Alleud, Belgium

**Keywords:** 2D-DIGE, design of experiments, hierarchical clustering, multiple linear regression, bioenergetics, metabolic network, biological system, environment

## Abstract

Microalgae are currently emerging to be very promising organisms for the production of biofuels and high-added value compounds. Understanding the influence of environmental alterations on their metabolism is a crucial issue. Light, carbon and nitrogen availability have been reported to induce important metabolic adaptations. So far, the influence of these variables has essentially been studied while varying only one or two environmental factors at the same time. The goal of the present work was to model the cellular proteomic adaptations of the green microalga *Chlamydomonas reinhardtii* upon the simultaneous changes of light intensity, carbon concentrations (CO_2_ and acetate), and inorganic nitrogen concentrations (nitrate and ammonium) in the culture medium. Statistical design of experiments (DOE) enabled to define 32 culture conditions to be tested experimentally. Relative protein abundance was quantified by two dimensional differential in-gel electrophoresis (2D-DIGE). Additional assays for respiration, photosynthesis, and lipid and pigment concentrations were also carried out. A hierarchical clustering survey enabled to partition biological variables (proteins + assays) into eight co-regulated clusters. In most cases, the biological variables partitioned in the same cluster had already been reported to participate to common biological functions (acetate assimilation, bioenergetic processes, light harvesting, Calvin cycle, and protein metabolism). The environmental regulation within each cluster was further characterized by a series of multivariate methods including principal component analysis and multiple linear regressions. This metadata analysis enabled to highlight the existence of a clear regulatory pattern for every cluster and to mathematically simulate the effects of light, carbon, and nitrogen. The influence of these environmental variables on cellular metabolism is described in details and thoroughly discussed. This work provides an overview of the metabolic adaptations contributing to maintain cellular homeostasis upon extensive environmental changes. Some of the results presented here could be used as starting points for more specific fundamental or applied investigations.

## Background

Freshwater green microalgae are known to undergo global metabolic reorganizations to adapt to changing environmental conditions. This enables microalgae to maintain their cellular homeostasis despite the onset of very dynamic modifications of physico-chemical parameters such as temperature, nutrient availability, or gas partial pressures (Falkowski and Raven, 2013). *Chlamydomonas reinhardtii* is a model organism which is commonly used to study photosynthetic processes. This green microalga exhibits a much faster growth rate than higher plants, is of easier maintenance and can be cultured under very diverse experimental conditions (Harris, 2001). *C. reinhardtii* is able to grow either in the light (photoautotrophy in the presence of CO_2_; mixotrophy in the presence of CO_2_ + organic carbon) or in the dark when an oxidizable carbon source is available in the medium (Spalding, 2009; Perez-Garcia et al., 2011). Moreover it can assimilate different chemical forms of nitrogen, either inorganic (nitrate, nitrite, ammonium) or organic (urea, amino acids, purine nucleotides; Fernandez et al., 2004). In 2007, the sequencing of the *C. reinhardtii* genome opened the gate to further characterization by a growing panel of molecular techniques such as targeted mutagenesis, transcriptomics and proteomics (Merchant et al., 2007).

Over the last decades, the influence of environmental changes on biological functions has been extensively studied in photosynthetic organisms. Light, carbon and nitrogen have been reported to induce dramatic metabolic adaptations as a way to maintain a proper bioenergetic balance. These adaptations can occur at very different levels such as genetic expression, protein abundance, enzymatic activity, or cellular structure (Tobin and Silverthorne, 1985; Spalding et al., 2002; Fernandez et al., 2004). To date, most studies have focused on the individual effects of light, carbon and nitrogen (light + carbon or nitrogen + carbon in a few cases). However, from available data, it is clear that their signaling and assimilatory pathways are connected through a complex metabolic network (Turpin, 1991; Huppe and Turpin, 1994; Singh et al., 2008). Understanding how photosynthetic organisms adapt to global environmental modifications could therefore be of prime interest. This is all the more true that green microalgae are currently emerging as very promising sources for the production of biofuels and high-added value compounds (Work et al., 2012). In this context, the bioenergetic adaptations of *C. reinhardtii* cells upon simultaneous changes related to light, carbon and inorganic nitrogen have recently been modeled by our group (Gérin et al., 2014). This work was carried out through a statistical approach coupling design of experiments (DOE) to multiple linear regression analyses. It enabled to build empirical models simulating mathematically the influence of each environmental variable and highlighting significant interactions between them in some cases.

Comparative proteomics is a suitable tool to characterize the metabolic adaptations induced by diverse endogenous or exogenous perturbations such as genetic modifications, pathologies, heat shocks or nutrient changes. In this field, proteomics is often preferred to transcriptomics since the correlation between mRNA abundance and protein expression is relatively weak, as reviewed in (Greenbaum et al., 2003) for yeast. 2D-DIGE (two dimensional differential in-gel electrophoresis) is a comparative proteomic technique requiring the pre-electrophoretic labeling of protein samples with three spectrally-distinct fluorescent dyes. Its capacity for multiplexing enables to introduce an internal standard in each gel electrophoresis to normalize protein abundance. Together with the relatively wide dynamic range of 2D-DIGE, this makes it possible to quantify very accurate changes in protein abundance (Marouga et al., 2005).

To date, an overview of the influence of cumulative environmental changes on photosynthetic metabolism is lacking. Some pathways have nevertheless been reported (mostly through univariate studies) to be regulated by two or several factors: see for example the well-known regulation of photosynthetic antennae size and pigment content by light and acetate, and the respective effects of acetate and inorganic nitrogen on the TCA cycle (Neale and Melis, 1986; Falkowski and LaRoche, 1991; Turpin, 1991; Huppe and Turpin, 1994; Teramoto et al., 2002; Durnford et al., 2003; Nield et al., 2004; Boyle and Morgan, 2009; Gérin et al., 2010, 2014). In the present work, we aimed to build statistical models describing the global metabolic adaptations of *C. reinhardtii* cells upon simultaneous changes of several environmental variables: light intensity, carbon concentration (acetate and CO_2_) and inorganic nitrogen concentration (nitrate and ammonium). Such an empirical approach appeared to us as a crucial pre-requisite before attempting to build mechanistic models in subsequent studies. For this purpose, 2D-DIGE was coupled to DOE and multivariate data analyses in order to characterize the environmental regulation of protein abundance at the cellular level. Additional assays for respiration, photosynthesis and cellular contents of some lipids and pigments were also carried out to this end. Hierarchical clustering was first performed to partition biological variables (proteins and assays) into discrete co-regulated clusters. The pattern of environmental regulation was then characterized within each cluster through a panel of multivariate statistical methods, including principal component analysis and multiple linear regressions. Overall, the data reported here provide an overview of the metabolic adaptations set up in response to global environmental changes related to light, carbon and inorganic nitrogen in *C. reinhardtii*.

## Material and methods

### Cell cultures

A *cw15* mt^+^ wall-less strain of *C. reinhardtii* (Hyams and Davies, 1972) was used in this study (*Chlamydomonas* Resource Center ID: CC-400). Algal cells were cultivated in lab-scale tubular photobioreactors (Multi-Cultivators MC 1000, Photon System Instruments) as described in Gérin et al. (2014), same media, conditions and procedures. Culture media invariably contained MgSO_4_ 1.4 mM, CaCl_2_ 450 μM, K_2_HPO_4_ 5.4 mM, KH_2_PO_4_ 4.6 mM, Tris-HCl 20 mM pH 7.2, added with oligo-elements (composition described in Gérin et al., 2014). When applicable according to the DOE, acetic acid (0–1 g.L^−1^), NaNO_3_ (0–20 mM) and NH_4_Cl (0–15 mM) were also added. CO_2_ was bubbled in the cultures at either 0.035% (ambient air) or 1.5% (mix of ambient air + pure CO_2_). Light intensity was tuned from 0 to 200 μmol_photons_.m^−2^.s^−1^ with the Multi-Cultivator interface. Algae were harvested by centrifuging at 3000 g for 5 min, washed in one volume of ice-cold saline buffer (NaCl 150 mM, Tris-HCl 50 mM, pH 7.2), centrifuged again in the same conditions and stored as pellets at −80°C for analyses.

### 2D-DIGE

#### Protein extraction and purification

Algal pellets were resuspended in an ice-cold extraction buffer (NaCl 150 mM, Triton X-100 0.1% (v/v), EDTA 1 mM, DL-dithiothreitol (DTT) 25 mM, complete EDTA-free protease inhibitor cocktail tablets (Roche), Tris-HCl 50 mM pH 7.8) added with polyvinylpolypyrrolidone (PVPP, insoluble in water) 2.5% (w/v) to complex polyphenols. Proteins were extracted by sonicating at 6 Amp for 30 s on ice (Sonifier Cell Disruptor B-12, Branson), vortexing for 30 s at 4°C, and repeating the procedure twice more. Protein extracts were centrifuged at 3000 g for 3 min at 4°C to spin down PVPP. The supernatant was centrifuged again at 10,000 g for 3 min to spin down cellular debris, and was then filtered with a 0.22 μm cellulose acetate-membrane syringe filter. Proteins were further purified according to the phenol phase separation procedure described by Carpentier et al. (2005), and were finally solubilized in an appropriate volume of a DIGE labeling buffer (urea 7 M, thiourea 2 M, ASB-14 2% (w/v), EDTA 0.5 mM, DTT 10 mM, Tris-HCl 50 mM pH 8.5) so as to reach a concentration comprised between 5 and 10 mg.mL^−1^.

#### Protein labeling

Protein samples were labeled with Refraction-2D G-Dyes from NH DyeAgnostics (May et al., 2012) and allocated to 16 different 2D-electrophoreses as detailed in Additional file 1. Each electrophoresis comprised two algal culture samples labeled with G-Dye200 and G-Dye300, and an internal standard (equal amount of all available samples) labeled with G-Dye100. In each case, 25 μg of proteins were labeled with 0.2 nmol of G-Dye for 30 min at 25°C in the dark. Labeling reactions were stopped by adding 1 μL of Stop Solution (DyeAgnostics) and incubating samples for 10 min in the same conditions. For preparative electrophoreses, a 500 μg pool of all samples in an equal amount was constituted, out of which 25 μg were labeled with G-Dye100 before being re-incorporated among the remaining 475 μg.

#### 2D-electrophoreses and image acquisition

Isoelectrofocusing (IEF) was carried out as previously reported (Mathy et al., 2010) by using a 3-11 non-linear pH range, except that the rehydration buffer was added with CHAPS 3% (w/v). IPG strips (GE Healthcare) were then rinsed with milliQ water before being reduced, alkylated and loaded on the top of polyacrylamide gels for SDS-PAGE separation as also described in this previous publication (Mathy et al., 2010), with the difference that 10% polyacrylamide gels (37.5:1 acrylamide-to-bisacrylamide ratio) were rather used in the present study. Images of G-Dyes within 2D-gels were acquired with a Typhoon 9400 scanner (GE Healthcare) by using the specific excitation and emission wavelength of each dye.

#### Image analysis

Images were analyzed with the DeCyder 7.0 software from GE Healthcare. Spot detection was performed in the Differential In-Gel Analysis (DIA) module with an exclusion filter restricting detection to protein spots with a volume superior or equal to 300,000 (for at least one G-Dye over three). The Biological Variation Analysis (BVA) module was then used to perform inter-gel matching of protein spots (Match Table) and to extract the abundance normalized by the internal standard for each spot and each culture condition (Appearance Table). These abundance values are the raw data used in the present statistical analyses.

#### Spot picking and protein identification by mass spectrometry

Protein spots were picked off preparative gels with an Ettan DALT Spot Picker device (GE Healthcare) and in-gel digested according to Shevchenko and co-workers (Shevchenko et al., 1996). Peptides were then extracted from gel pieces and prepared for mass spectrometry as previously described (Mathy et al., 2010).

Acquisition of mass spectra was carried out with a MALDI-TOF/TOF mass spectrometer (Ultraflex II, Bruker Daltonics) in PMF mode. The device was piloted by FlexControl 3.0, with real-time analysis of mass spectra by FlexAnalysis 3.0 and database search by BioTools 3.1 in the Mascot server, version 2.2.04. Database search was performed in NCBInr restricted to *Viridiplantae* (1,930,642 sequences) with the Mascot PMF algorithm as search engine and 100 ppm of mass error tolerance. Cysteine carbamidomethylation and methionine oxidation were assessed as fixed and variable peptide modifications, respectively. Protein identification was considered as successful for Mascot scores equal or superior to 75. Protein function(s) and cellular location(s) were searched in the ChlamyCyc database, version 1.0 (May et al., 2009).

### Pigment extraction and analysis

For the determination of chlorophyll *a*, chlorophyll *b* and total carotenoid (TC) contents, the absorbance of pigment extracts in methanol was measured at 470, 652, and 665 nm, and the Lichtenthaler and Wellburn's formulas (Lichtenthaler and Wellburn, 1983) were applied.

Neoxanthin, lutein, violaxanthin, and β-carotene concentrations were determined by high pressure liquid chromatography (HPLC) by using pigment extracts in methanol. Pigments were separated in a Nova-Pak silica-based, reverse-phase, 4 μm particle C_18_ column (Waters, product WAT036975, length: 150 mm, inner diameter: 3.9 mm). HPLC experiments were run with a 1 mL.min^−1^ flow rate at 25°C with three working solutions: solution A (methanol 90%, ammonium acetate 100 mM), solution B (acetonitrile 90%), and solution C (ethyl acetate 100%). The following protocol was applied as gradient: 0 min–100% A; 0.5 min–100% B; 1.1 min–90% B + 10% C; 6.1 min–65% B + 35% C; 11.5 min–40% B + 60% C; 15.0 min–100% C; 17.0 min–100% A; 23.0 min–100% A. Pigment elution times were determined by using the Mixed phytoplankton pigment standard (PPS-MIX-1) from DHI Lab Products. Chromatograms were analyzed at 430 nm and relative pigment concentrations were assessed in terms of peak areas at this wavelength.

### Fatty acid extraction and analysis

Fatty acids were extracted with chloroform-methanol and transesterified as previously described (Bligh and Dyer, 1959; Browse et al., 1986). Fatty acid concentrations were determined by gas chromatography (GC) with a BPX70 70% cyanopropylpolysilphenylene-siloxane column (SGE Analytical Science, product 054622, length: 2 m, inner diameter: 0.25 mm) with helium as carrier gas at 250°C. FAMES elution times and calibration curve were determined by running the Supelco 37 Component FAMES mix standard from Sigma-Aldrich (product CRM47885).

### Triglyceride extraction and analysis

Algal pellets were resuspended in an extraction buffer [NaCl 150 mM, Triton X-100 0.1% (v/v), Tris-HCl 50 mM pH 7.5], sonicated at 3 Amp for 15 s (Sonifier Cell Disruptor B-12, Branson) and thoroughly vortexed for 20 min. Triglyceride concentration was determined by using the enzymatic assay kit of BioVision (product K622-100).

### Protein assay for 2D-DIGE and pigment/lipid normalization

Protein concentrations were determined by using the Reagent Compatible/Detergent Compatible assay kit from BioRad (product 500–0121) which is based on the Lowry-Ciocalteu colorimetric method (Lowry et al., 1951).

### Respiratory and photosynthetic parameters

Bioenergetic data were extracted from our previous modeling publication (Gérin et al., 2014). As described there, these data were obtained by oxymetric measurements and pulse-amplitude-modulated (PAM) fluorimetry. Photosynthetic parameters (φPSII_800_, P_800_, and NPQ_800_) were measured under a saturating light irradiance of 800 μmol_photons_.m^−2^.s^−1^.

### Statistical analyses

#### Design of experiments

Design of experiments (DOE) was carried out with the Custom design platform of the JMP 11 software (SAS) with the following parameters: one dependent variable (goal: none); five environmental variables (changes: easy); single effects, 2nd-degree polynomial effects (for continuous factors), and 2nd-order interactions (estimability: necessary); 7 center points, zero replicate runs, default number of assays; randomize output order. Light intensity, nitrogen concentrations, and acetate concentration were considered as continuous variables, whereas CO_2_ concentration was considered as an ordinal variable with two modalities. For each continuous environmental variable, the minimal value was set to zero. The maximal values (described earlier) were chosen as follow:

- for nitrogen and acetate concentrations: twice higher than the optimal level—this generates values that enable biomass accumulation but remain below toxicity (Sager and Granick, 1953; Chen and Johns, 1994, 1996; Collos and Harrison, 2014; Gérin et al., 2014).
- for light intensity: insufficient to saturate the photosynthetic apparatus in order to limit photo-oxidative damages during algal cultivation (Sueltemeyer et al., 1986; White and Critchley, 1999).

As described above, the two modalities of CO_2_ concentration were set at its atmospheric level (0.035%) and at a saturating concentration (1.5%) sufficient to ensure no CO_2_ limitation for RubisCO whatever the light intensity (Vance and Spalding, 2005).

#### Initial screening for biological variables

An initial screening of the biological variables relevantly influenced by one or several environmental variable(s) was carried out with the JMP 11 software (SAS) by a methodology coupling PLSR and MLR as detailed in Table 1. PLSRs were run in the Mulivariate methods platform through the NIPALS algorithm with selection of the Centering and Scaling options. Leave-one-out validation method was chosen, and the default factor search range displayed by the software was not modified. The optimal number of latent factors was determined by using minimal PRESS (prediction error sum of squares) coupled to van der Voet *T*^2^ tests as selection criteria. MLRs were run in the Fit model platform. Screening was performed independently for protein spots and additional assays.

**Table 1:** **Input parameters and selection criteria used for the initial screening of the biological variables**.

	**PLSR**	**MLR**
	**One model per continuous environmental Variable**	
	**Strategy 1**	**Strategy 2**
JMP input (launch panel parameters)	Responses = all biological variables (proteins or assays)	Responses = all biological variables (proteins or assays)
	Factors = Acetate, Light, NH_4_, or NO_3_	Factors = Acetate, Light, NH_4_, or NO_3_
	By: CO_2_	Continuous environmental variable^2^
	Factor search range = 1	Continuous environmental variable × CO_2_
Number of models	8 models (optimal number of latent factors = 1)	4 models
Selection criterion for the biological variables	≥ 30% of variability explained by the latent factor for at least 1 model over 8 (≥ 19% in the NO_3_ models for protein spots)	Statistical significance with *p* ≤ 0.075 for at least 1 model over 4 for protein spots/*p* ≤ 0.05 for additional assays
	**Unique model with all continuous environmental variables**	
	**Strategy 3**	**Strategy 4**
JMP input (launch panel parameters)	Responses = all biological variables (proteins or assays)	Responses = all biological variables (proteins or assays)
	Factors = Acetate, Light, NH_4_, and NO_3_	Factors = Acetate, Light, NH_4_, and NO_3_
	By: CO_2_	Acetate^2^, Light^2^, ${\text{NH}}_{4}^{2}$, and ${\text{NO}}_{3}^{2}$
	Factor search range = 4	Acetate × CO_2_, Light × CO_2_, NH_4_ × CO_2_, and NO_3_ × CO_2_
Number of models	2 models (optimal number of latent factors = 2 for protein spots; 4 for additional assays)	1 model
Selection criterion for the biological variables	≥30% of variability explained by the latent factors in at least 1 model over 2 for protein spots/ = 65% for additional assays	Statistical significance of the model with *p* ≤ 0.1 for protein spots/*p* ≤ 0.05 for additional assays
	Selection of the biological variables encountering the selection criterion for at least 3 strategies over 4	

#### Hierarchical clustering

Hierarchical clustering was performed in the Multivariate methods platform of the JMP 11 software (SAS) by the Ward's minimum variance method (Ward, 1963; SAS, 2013). The options “Standardize data” and “Missing value imputation” were selected. The imputation of missing values was performed as follow: a single covariance matrix was built by the pairwise method on the basis of the whole data set; the non-missing variables were then used as predictors to impute missing values by a method equivalent to regression prediction (SAS, 2013).

#### Gene set enrichment analyses

Gene set enrichment analyses were performed in the PANTHER (Protein ANalysis THrough Evolutionary Relationships) database. The GI numbers in NCBI were used as protein IDs. *Chlamydomonas reinhardtii* was selected as organism. The “PANTHER Overrepresentation test (release 20160321)” was used as analysis type. The annotation data set was either “PANTHER Pathways” (PANTHER version 10.0 Released 2015-05-15) or “GO cellular component complete” (GO Ontology database Released 2016-05-20). The *p*-values were extracted with and without Bonferroni correction for multiple testing.

#### Principal component analysis (PCA) and in-cluster PLSRs

These procedures were both performed in the Multivariate methods platform of the JMP 12 software (SAS) with standardized data (i.e., data scaled to a mean of 0 and centered to a variance of 1 for each biological and environmental variable). PCA was carried out by the pairwise method on the basis of the correlation matrix with all biological variables in the same PCA. In-cluster PLSRs were performed through the NIPALS algorithm with all five environmental variables as factors. For CO_2_ concentration, data were first transformed according to a binary code: 0 for the lowest modality (0.035% CO_2_) and 1 for the highest one (1.5% CO_2_). Leave-one-out validation method was chosen, and the default factor search range displayed by the software was set to five. The optimal number of latent factors was determined by using minimal PRESS (prediction error sum of squares) coupled to van der Voet *T*^2^ tests as selection criteria. All biological variables (proteins + assays) belonging to each cluster were included in the same PLSR analysis.

#### Multiple correlations

Multiple correlations were assessed in the Multivariate methods platform of the JMP 11 software (SAS). The Pearson's correlation coefficients (R) between biological variables and the corresponding *p*-values were calculated by the pairwise method without missing value imputation. Data were previously centered to a mean of 0 and scaled to a variance of 1 for every biological variable before being analyzed.

#### Modeling the dependence of biological variables upon environmental variables

Modeling was performed in the Fit model platform of the JMP 11 software (SAS) on the basis of raw data listed in Additional file 2, following the same procedure as previously described (Gérin et al., 2014). Linear effects, quadratic effects and second-order interactions of the environmental variables were assumed.

##### Stepwise regression

Stepwise regression was carried out in forward direction with minimum AICc (corrected Akaike information criterion) as stopping rule (Burnham and Anderson, 2004).

##### Multiple linear regression (MLR)

MLR modeling was performed with the stepwise-selected effects by adjusting the coefficients of the following type of equation:

$$\begin{array}{l} \hat{y}=b_{0}+\sum b_{i}x_{i}+\sum b_{ii}x_{i}^{2}+\sum b_{ij}x_{i}x_{j}+b_{CO2}\\ \;+\sum b_{iCO2}x_{i}+e \end{array}$$

where ŷ is the predicted value of the biological variable, *b*_**0**_ the intercept and *e* the residual. Continuous environmental variables are designated by *x*_*i*_ or *x*_*j*_, and their linear, quadratic and interaction coefficients are pointed out as *b*_*i*_, *b*_*ii*_, and *b*_*ij*_, respectively. The coefficients related to CO_2_ concentration (ordinal variable) enable to characterize the modification of ŷ while switching from the lowest modality (0.035% CO_2_) to the highest one (1.5% CO_2_). *b*_*CO*2_ is for the single effect of CO_2_ concentration, whereas *b*_*iCO*2_ designates the interactions of CO_2_ with continuous environmental variables.

The goodness of fit of the models was assessed by calculating the coefficients of multiple determination (*R*^2^ and *R*^2^ adjusted) and the fitting root-mean-squared error (*RMSE*_*F*_) as follow:

$$\begin{array}{l} \;R^{2}=\frac{\sum \;{\left({\hat{y}}_{i}-\bar{y}\right)}^{2}}{\sum \;{\left(y_{i}-\bar{y}\right)}^{2}}\\ R^{2}\;adjusted=1-\frac{\sum \;{\left(y_{i}-{\hat{y}}_{i}\right)}^{2}/\;(n-k-1)}{\sum \;{\left(y_{i}-\bar{y}\right)}^{2}/\;(n-1)}\\ \;RMSE_{F}=\sqrt{\frac{\sum \;{\left(y_{i}-{\hat{y}}_{i}\right)}^{2}}{n-k-1}} \end{array}$$

where *n* and *k* are the number of observations and coefficients (apart from *b*_**0**_) within the model, respectively, *y*_*i*_ and ŷ_*i*_ are the observed and predicted values of the biological variable, respectively, and ȳ is the experimental mean value of the biological variable. The average scale of each biological variable was calculated as follow and exhibited in parallel to *RMSE*_*F*_ as a reference to assess the extent of the deviations:

$$Response\;average\;scale=\bar{y}-y_{MIN}$$

where ȳ and *y*_*MIN*_ are the mean and minimal experimental values of the biological variable, respectively.

The statistical significance of the models was assessed by calculating whole-model ANOVA tests with the following expression for the *F*-ratio:

$$F_{whole-model}=\frac{\sum \;{\left({\hat{y}}_{i}\;-\;\bar{y}\right)}^{2}/\;k}{\sum \;{\left(y_{i}-{\hat{y}}_{i}\right)}^{2}/\;(n-k-1)}$$

where the terms are the same than those described for *R*^2^, *R*^2^ adjusted, and *RMSE*_*F*_ (cutoff for statistical significance: *p* ≤ 0.05).

The importance and statistical significance of each individual effect of the environmental variables were assessed by calculating the related β-weights (= standardized regression coefficients) and ANOVA tests, respectively. For the latter tests, the *F*-ratio was calculated as follow:

$$F_{effect}=\frac{\sum {\left(y_{i}-{\hat{y}}_{i\;(k-1)}\right)}^{2}\;-\;\sum \;{\left(y_{i}-{\hat{y}}_{i}\right)}^{2}}{\sum \;{\left(y_{i}-{\hat{y}}_{i}\right)}^{2}\;/\;(n-k-1)}$$

where the terms with an “*i*” subscript, *n* and *k* have the same significance than described above whereas ŷ_*i* (*k*−1)_ points out the predicted values of the biological variable in a hypothetical model deprived of the effect (cutoff for statistical significance: *p* ≤ 0.05).

Lack-of-fit ANOVA tests were carried out to assess whether the models were lacking one or several major explanatory effect(s) (cutoff for statistical significance: *p* ≤ 0.05). The *F*-ratio was calculated as the quotient between the mean square for lack-of-fit error and the mean square for pure error (for details see SAS, 2012).

#### Model cross-validation

Models were cross-validated by the *k*-fold method (*k* = 4) with the Statistical 10 software (StatSoft) by using the data subsets defined in Additional file 2. The goodness of fit of the training models was assessed by calculating *R*^2^, *R*^2^ adjusted, and *RMSE*_*F*_ (read above for details about calculations). The deviation of each validation data set from its corresponding training model was assessed in terms of cross-validation root-mean-squared error (*RMSE*_*CV*_), which was calculated as follow:

$$RMSE_{CV}=\sqrt{\frac{\sum {\left(y_{v}-{\hat{y}}_{v}\right)}^{2}}{v}}$$

where *y*_*v*_ are the observed values for the validation data set, ŷ_*v*_ the values predicted by the training model for the validation data set and *v* is the number of observations in the validation data set.

#### Analysis of covariance (ANCOVA)

Analysis of covariance (ANCOVA) was performed through MLR on the basis of standardized data (i.e., data scaled to a mean of 0 and a variance of 1) independently within each cluster. The following general equation was used:

$$\begin{array}{l} \hat{y}=b_{0}+\sum b_{i}x_{i}+\sum b_{ii}x_{i}^{2}+\sum b_{ij}x_{i}x_{j}+b_{CO2}\\ \;+\sum b_{iCO2}x_{i}+e+\sum b_{m}+\sum b_{mi}x_{i}+\sum b_{mCO2} \end{array}$$

in which the identity of the biological variables is a categorical predictor with *n* modalities (*m*_**1**_*, m*_**2**_*, …, m*_*n*_), *b*_**0**_ is the intercept and *e* the residual. The terms which are not highlighted in bold concern environmental factors alone (read above the section on MLR). The terms in bold refer to the single effects of the biological variables (*b*_*m*_) and to the second-order interactions between biological and environmental variables (*b*_*mi*_ *x*_*i*_ for acetate, light, nitrate, and ammonium; *b*_*mCO*2**_ for CO_2_). ANCOVA models were characterized by the same goodness of fit and statistical parameters than described above for MLR.

## Results

**Figure 1:**
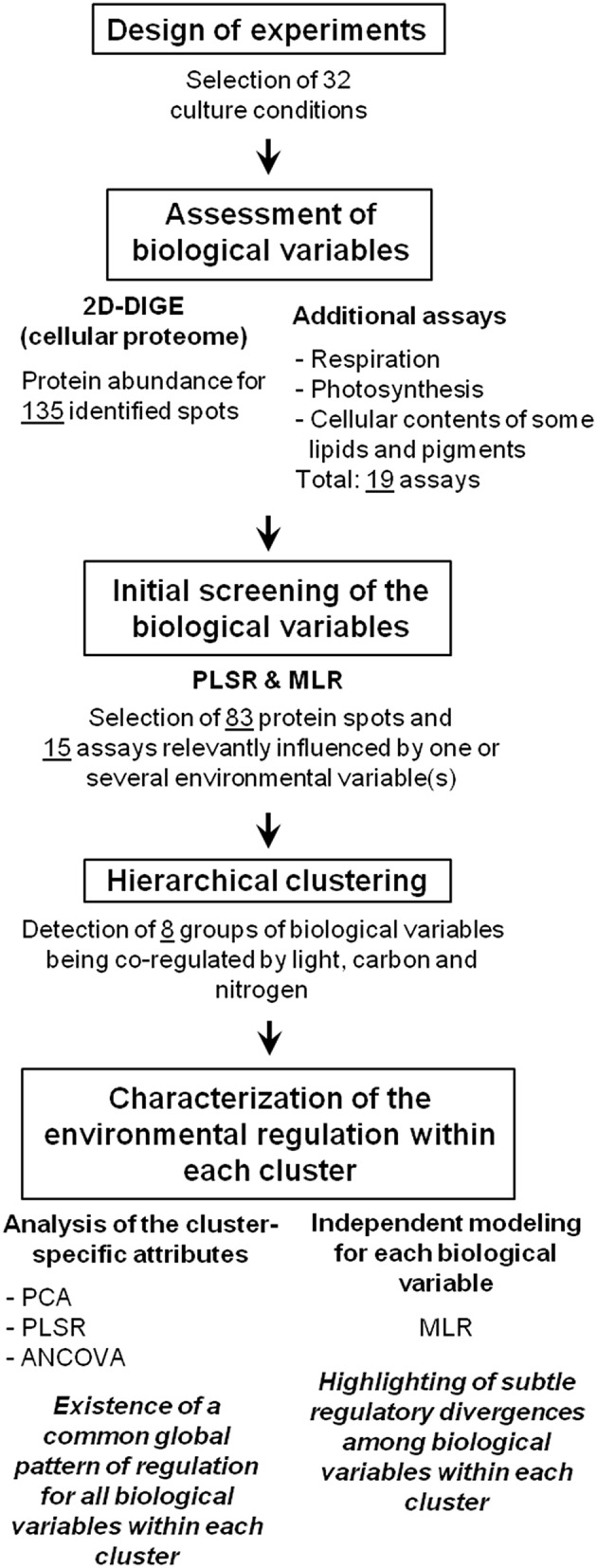
**Overview of the methodology and results of the present study**. PLSR, partial least squares regression; MLR, multiple linear regression; PCA, principal component analysis; ANCOVA, analysis of covariance.

A step-by-step overview of the methodology and results is presented in Figure 1.

### Characterization of the environmental regulation of proteins and other biological variables through multivariate statistics

#### Environmental variables and design of experiments

Environmental variables are light intensity and acetate, CO_2_, nitrate, and ammonium concentrations in the culture medium (five variables in total). Their characteristics are summarized in Table 2 as the type of each variable, its working range (or modalities for CO_2_ concentration) and its unit (similar features as described in Gérin et al., 2014).

Table 2**Description of the environmental and biological variables considered in the present work**.

**Table:** 

**Environmental variables—Design of experiments**				
**Variables**	**Type**	**Unit**	**Range/Modalities**
Acetate concentration	Continuous	g.L^−1^	0–1
Light intensity	Continuous	μmol_photons_.m^−2^.s^−1^	0–200
Ammonium concentration	Continuous	mM	0–15
Nitrate concentration	Continuous	mM	0–20
CO_2_ concentration	Ordinal	%	0.035 and 1.5

**Table:** 

**Biological variables—Experimental determination**				
**Source**	**Variables**			**Unit**
2D-DIGE	Cellular abundance of…	All 135 identified protein spots (see Table 4)		Spot volume normalized by the I.S.
GC		Palmitic acid		μg.mg^−1^_proteins_
		Stearic acid		
		Oleic acid		
		γ-linolenic acid		
		Linolenic acid		
Enzymatic assay		Triglycerides		μg.mg^−1^_proteins_
Lichtenthaler's spectroscopic equations		Chlorophyll a		μg.mg^−1^_proteins_
		Chlorophyll b		
		Total carotenoids		
HPLC		Neoxanthin		Peak area.mg^−1^_proteins_
		Violaxanthin		
		Lutein		
		β-carotene		
Clark's electrode oxymetry	Data from Gérin et al., 2014 for…	CR	(Cellular respiration)	nmolO_2_.min^−1^.mg^−1^_proteins_
		MA_CYT_	(Apparent maximal activity of the cytochrome pathway)	
		MA_ALT_	(Apparent maximal activity of the alternative pathway)	
		P_800_	(Gross photosynthetic O_2_ evolution)	
PAM fluorimetry		φPSII_800_	(Quantum yield of photosystem II)	Arbitrary
		NPQ_800_	(Non-photochemical quenching of chlorophyll fluorescence)	

*Design of experiments (DOE) was carried out to determine the combinations of environmental variables for which the corresponding biological variables should be measured (see Additional file 2). For CR, MA_CYT_, MA_ALT_, P_800_, φPSII_800_, and NPQ_800_, data were collected from a recent publication from our group (Gérin et al., 2014) performed with the same algal strain and experimental conditions, and with a similar DOE*.

A statistical design of experiments (DOE) was built to define discrete combinations of the environmental variables to be tested experimentally. Linear effects, quadratic effects, and second-order interactions of the environmental variables were considered. The features of this DOE are similar to the design of our previous modeling study (Gérin et al., 2014): the DOE consists of a two level fractional factorial design added with center points and supplementary points found in the Box-Behnken and central composite types of designs. It contains 32 culture conditions, that were already found in the previous DOE (see Gérin et al., 2014 for more information). Table 3 displays a complete list of DOE items, and a 3D representative example of space covering by environmental variables for light, acetate and nitrate can be found in Additional file 2.

**Table 3:** **Design of experiments**.

**Identification number**	**[Acetate]**	**Light**	**[${\text{NH}}_{4}^{+}$]**	**[${\text{NO}}_{3}^{-}$]**	**[CO_2_]**
1	0	200	0	20	1.5
2	0	200	0	10	1.5
3	0.5	200	0	0	1.5
4	0.5	100	7.5	10	1.5
5	1	200	0	20	0.035
6	1	0	15	0	1.5
8	1	0	0	0	0.035
9	0	0	15	20	1.5
13	0	100	0	0	1.5
15	0	0	0	0	0.035
16	1	200	15	0	1.5
19	1	0	0	20	1.5
20	1	200	15	20	0.035
21	0.5	100	7.5	10	0.035
22	1	0	15	0	0.035
23	0	200	15	20	0.035
24	1	0	0	0	1.5
25	0	200	15	0	0.035
27	1	200	15	0	0.035
29	0	0	7.5	0	1.5
30	0	0	15	20	0.035
31	0	0	15	0	0.035
32	0	200	0	20	0.035
33	0.5	200	15	10	1.5
34	1	0	15	20	0.035
35	1	200	15	20	1.5
36	0	0	0	20	0.035
37	1	200	0	0	0.035
39	0.5	100	7.5	10	0.035
40	0	200	0	0	0.035
41	0.5	100	7.5	10	1.5
42	0.5	100	7.5	10	1.5

The identification number of each item refers to the DOE described in Gérin et al. (2014), which served as basis to build the present one. The unit of each environmental variable can be found in Table 2.

The environmental variables were tested for collinearity by calculating the Pearson's correlation coefficients (*R*) between them. No statistically significant correlation could be detected (*R* ≤ 0.16 with *p* ≥ 0.3908), indicating that the design space was uniformly covered.

#### Biological variables

##### 2D-DIGE

**Figure 2:**
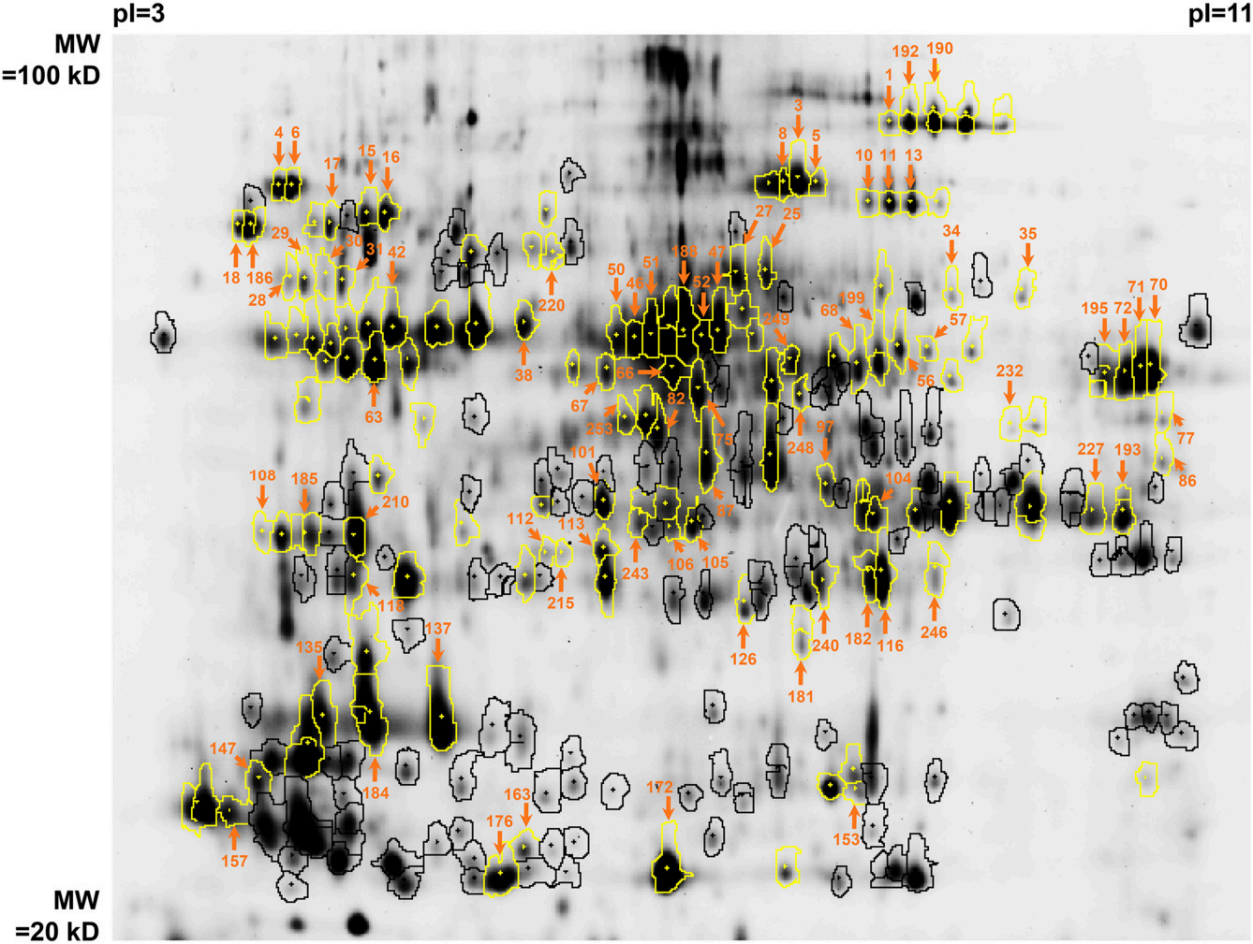
**Image of the G-Dye100-labeled internal standard in the Master gel (n°11 in Additional file 1)**. The spots which were detected by DeCyder 7.0 and which passed the volume restriction filter are encircled. Among them, those that could be identified by mass spectrometry are highlighted in yellow (see also Table 4). The spots that passed the initial PLSR- and MLR-based screening are pointed out by orange arrows with surrounding Master numbers (see also Additional file 3). pI, isoelectric point; MW, molecular weight.

The fluorescence image of the internal standard in the Master 2D-gel is presented in Figure 2. In order to minimize the experimental error of protein abundance estimation, the volume exclusion filter for the detection of protein spots was fixed at a tenfold-higher value than recommended by the manufacturer (GE Healthcare). This procedure led to detect 254 spots that could be matched among all 2D-gels (these spots are encircled in Figure 2). Among them, 135 could be identified by mass spectrometry. These spots of interest were defined as continuous biological variables for subsequent statistical analyses (Table 2). They are highlighted in yellow in Figure 2 and complete descriptions about them are provided in Table 4. For each of these spots and each DOE culture condition, the abundance value normalized by the internal standard value was extracted from the Appearance Table of the DeCyder 7.0 BVA module and considered as raw data for subsequent statistical analyses (Additional file 2). To facilitate data treatment and result description, we decided to designate protein spots by their Master number (i.e., their identifier in the Master 2D-gel) followed by their standard name in databases (as found in Table 4).

**Table 4:** **Results of mass spectrometry identifications**.

**Master number**	**Gene name**	**GI number in NCBI**	**Protein description**	**pI**	**MW**	**I.S. spot volume in Master gel**
**1**	**ACH1**	**gi|159462944**	**Aconitate hydratase**	**8.9**	**86754**	**140111**
**190**	**ACH1**	**gi|159462944**	**Aconitate hydratase**	**8.9**	**86754**	**855573**
**192**	**ACH1**	**gi|159462944**	**Aconitate hydratase**	**8.9**	**86754**	**459786**
**10**	**ACS3**	**gi|159488061**	**Acetyl CoA synthetase**	**7.3**	**74089**	**179390**
**11**	**ACS3**	**gi|159488061**	**Acetyl CoA synthetase**	**7.3**	**74089**	**352380**
**13**	**ACS3**	**gi|159488061**	**Acetyl CoA synthetase**	**7.3**	**74089**	**439169**
**66**	**AGS1**	**gi|159477301**	**Argininosuccinate synthase**	**8.4**	**49218**	**780829**
**67**	**AGS1**	**gi|159477301**	**Argininosuccinate synthase**	**8.4**	**49218**	**224729**
**220**	**ASA1**	**gi|159468466**	**Mitochondrial F1F0 ATP synthase associated 60.6 kDa protein**	**5.8**	**63123**	**173474**
**86**	**AST1**	**gi|159473837**	**Aspartate aminotransferase**	**9.7**	**46902**	**149498**
**18**	**ATP2**	**gi|159466892**	**Beta subunit of mitochondrial ATP synthase**	**5.0**	**61954**	**231602**
**186**	**ATP2**	**gi|159466892**	**Beta subunit of mitochondrial ATP synthase**	**5.0**	**61954**	**863821**
**38**	**ATPA**	**gi|41179050**	**ATP synthase CF1 alpha subunit**	**5.4**	**54832**	**495031**
**42**	**ATPA**	**gi|41179050**	**ATP synthase CF1 alpha subunit**	**5.4**	**54832**	**1711421**
**181**	**ATPvE**	**gi|159469570**	**Vacuolar ATP synthase subunit E**	**7.5**	**26399**	**230593**
**68**	**BCR1**	**gi|159488652**	**Biotin carboxylase, acetyl-CoA carboxylase component**	**9.0**	**52308**	**494766**
**199**	**BCR1**	**gi|159488652**	**Biotin carboxylase, acetyl-CoA carboxylase component**	**9.0**	**52308**	**493171**
**63**	**BLD10**	**gi|159489304**	**Basal body protein**	**5.0**	**174819**	**3481530**
**56**	**CAT1**	**gi|159477329**	**Catalase/peroxidase**	**6.9**	**56407**	**397278**
**57**	**CAT1**	**gi|159477329**	**Catalase/peroxidase**	**6.9**	**56407**	**107595**
**232**	**CIS1**	**gi|159490012**	**Citrate synthase**	**9.1**	**51376**	**74708**
**28**	**CPN60A**	**gi|159491478**	**Chaperonin 60A**	**5.5**	**61911**	**146530**
**29**	**CPN60A**	**gi|159491478**	**Chaperonin 60A**	**5.5**	**61911**	**309631**
**30**	**CPN60A**	**gi|159491478**	**Chaperonin 60A**	**5.5**	**61911**	**113632**
**31**	**CPN60A**	**gi|159491478**	**Chaperonin 60A**	**5.5**	**61911**	**269507**
**126**	**CPX1**	**gi|159487437**	**Coproporphyrinogen III oxidase**	**9.0**	**41743**	**288842**
**108**	**CYN38**	**gi|159467709**	**Peptidyl-prolyl cis-trans isomerase, cyclophilin-type**	**5.4**	**44781**	**138802**
**248**	**CYP55B1**	**gi|159484456**	**Cytochrome P450, nitric oxide reductase**	**6.5**	**44185**	**210579**
**70**	**EEF1A1**	**gi|159476938**	**Eukaryotic translation elongation factor 1 alpha 1**	**8.7**	**51191**	**2335176**
**71**	**EEF1A1**	**gi|159476938**	**Eukaryotic translation elongation factor 1 alpha 1**	**8.7**	**51191**	**2072864**
**72**	**EEF1A1**	**gi|159476938**	**Eukaryotic translation elongation factor 1 alpha 1**	**8.7**	**51191**	**1071287**
**195**	**EEF1A1**	**gi|159476938**	**Eukaryotic translation elongation factor 1 alpha 1**	**8.7**	**51191**	**587077**
**253**	**EFTU_III**	**gi|41179007**	**Elongation factor Tu**	**5.9**	**45772**	**237513**
**116**	**FNR1**	**gi|159478523**	**Ferredoxin-nadp reductase**	**8.5**	**38698**	**1802268**
**182**	**FNR1**	**gi|159478523**	**Ferredoxin-nadp reductase**	**8.5**	**38698**	**538494**
**240**	**FNR1**	**gi|159478523**	**Ferredoxin-nadp reductase**	**8.5**	**38698**	**452769**
**246**	**FNR1**	**gi|159478523**	**Ferredoxin-nadp reductase**	**8.5**	**38698**	**204137**
**17**	**FTSH1**	**gi|159465357**	**Membrane AAA-metalloprotease**	**5.6**	**77727**	**282826**
**147**	**FTT2**	**gi|159477028**	**14-3-3 protein**	**4.9**	**28099**	**389454**
**193**	**GAP3**	**gi|159463282**	**Glyceraldehyde-3-phosphate dehydrogenase**	**9.2**	**40507**	**570521**
**227**	**GAP3**	**gi|159463282**	**Glyceraldehyde-3-phosphate dehydrogenase**	**9.2**	**40507**	**557330**
**105**	**GLN2**	**gi|159469782**	**Glutamine synthetase**	**7.1**	**41715**	**310080**
**106**	**GLN2**	**gi|159469782**	**Glutamine synthetase**	**7.1**	**41715**	**155830**
**243**	**GLN2**	**gi|159469782**	**Glutamine synthetase**	**7.1**	**41715**	**335820**
**163**	**GSTS2**	**gi|159482414**	**Glutathione S-transferase**	**5.5**	**23922**	**285290**
**15**	**HSP70A**	**gi|159486599**	**Heat shock protein 70A**	**5.3**	**71513**	**468194**
**16**	**HSP70A**	**gi|159486599**	**Heat shock protein 70A**	**5.3**	**71513**	**611102**
**4**	**HSP70B**	**gi|159476666**	**Heat shock protein 70B**	**5.2**	**72081**	**502751**
**6**	**HSP70B**	**gi|159476666**	**Heat shock protein 70B**	**5.2**	**72081**	**872383**
**82**	**ICL1**	**gi|159474436**	**Isocitrate lyase**	**5.9**	**45948**	**643018**
**104**	**IDH2**	**gi|159473471**	**Isocitrate dehydrogenase–NAD-dependent**	**8.8**	**38796**	**388985**
**118**	**LHCB5**	**gi|159475641**	**Minor chlorophyll a-b binding protein of photosystem II**	**5.4**	**30695**	**608401**
**157**	**LHCBM1**	**gi|20269804**	**Major light-harvesting complex II protein m1**	**6.0**	**27605**	**396217**
**135**	**LHCBM3**	**gi|159491492**	**Light-harvesting complex II chlorophyll a-b binding protein M3**	**5.7**	**27420**	**1168663**
**184**	**LHCBM3**	**gi|159491492**	**Light-harvesting complex II chlorophyll a-b binding protein M3**	**5.7**	**27420**	**3613827**
**137**	**LHCBM6**	**gi|159474480**	**Chloropyll a-b binding protein of LHCII type I, chloroplast precursor**	**5.9**	**27058**	**3345291**
**34**	**MAS1**	**gi|159475042**	**Malate synthase**	**8.7**	**61011**	**126787**
**35**	**MAS1**	**gi|159475042**	**Malate synthase**	**8.7**	**61011**	**149623**
**75**	**METM**	**gi|159477124**	**S-Adenosylmethionine synthetase**	**6.0**	**43070**	**647025**
**77**	**MPPA2**	**gi|159465665**	**Mitochondrial processing peptidase alpha subunit**	**9.7**	**49559**	**122510**
**25**	**PCK1a**	**gi|159473685**	**Phosphoenolpyruvate carboxykinase - splice variant a**	**6.2**	**62388**	**342592**
**27**	**PCK1a**	**gi|159473685**	**Phosphoenolpyruvate carboxykinase - splice variant a**	**6.2**	**62388**	**568919**
**87**	**PGK1**	**gi|159482940**	**Phosphoglycerate kinase**	**8.9**	**49172**	**913779**
**153**	**POA1**	**gi|159467074**	**20S proteasome alpha subunit A**	**7.6**	**27487**	**199430**
**101**	**PRK1**	**gi|159471788**	**Phosphoribulokinase**	**9.0**	**42151**	**704307**
**172**	**PSBP1**	**gi|159471964**	**Oxygen-evolving enhancer protein 2 of photosystem II**	**9.8**	**29971**	**6598334**
**176**	**PSBP1**	**gi|159471964**	**Oxygen-evolving enhancer protein 2 of photosystem II**	**9.8**	**29971**	**1332361**
**46**	**RBCL**	**gi|41179049**	**Ribulose-1,5-bisphosphate carboxylase/oxygenase large subunit**	**6.1**	**53193**	**768686**
**47**	**RBCL**	**gi|41179049**	**Ribulose-1,5-bisphosphate carboxylase/oxygenase large subunit**	**6.1**	**53193**	**1962505**
**50**	**RBCL**	**gi|41179049**	**Ribulose-1,5-bisphosphate carboxylase/oxygenase large subunit**	**6.1**	**53193**	**573314**
**51**	**RBCL**	**gi|41179049**	**Ribulose-1,5-bisphosphate carboxylase/oxygenase large subunit**	**6.1**	**53193**	**2761041**
**52**	**RBCL**	**gi|41179049**	**Ribulose-1,5-bisphosphate carboxylase/oxygenase large subunit**	**6.1**	**53193**	**953428**
**188**	**RBCL**	**gi|41179049**	**Ribulose-1,5-bisphosphate carboxylase/oxygenase large subunit**	**6.1**	**53193**	**11015508**
**185**	**RPSA**	**gi|159489000**	**Ribosomal protein Sa, component of cytosolic 80S ribosome and 40S small subunit**	**5.1**	**30971**	**447905**
**210**	**SEBP1**	**gi|159467635**	**Sedoheptulose-1,7-bisphosphatase**	**9.6**	**42393**	**1334623**
**249**	**SHMT2**	**gi|159487140**	**Serine hydroxymethyltransferase 2**	**6.3**	**52228**	**219816**
**97**	**SNE5**	**gi|159487407**	**NAD-dependent epimerase/dehydratase**	**7.8**	**36568**	**211419**
**3**	**TRK1**	**gi|159487741**	**Transketolase**	**7.1**	**78352**	**1478739**
**5**	**TRK1**	**gi|159487741**	**Transketolase**	**7.1**	**78352**	**216394**
**8**	**TRK1**	**gi|159487741**	**Transketolase**	**7.1**	**78352**	**470044**
**112**	**UPTG1**	**gi|159471081**	**UDP-Glucose:protein transglucosylase**	**5.9**	**39846**	**91980**
**113**	**UPTG1**	**gi|159471081**	**UDP-Glucose:protein transglucosylase**	**5.9**	**39846**	**348353**
**215**	**UPTG1**	**gi|159471081**	**UDP-Glucose:protein transglucosylase**	**5.9**	**39846**	**68875**
191	ACH1	gi|159462944	Aconitate hydratase	8.9	86754	783724
231	ACH1	gi|159462944	Aconitate hydratase	8.9	86754	164370
12	ACS3	gi|159488061	Acetyl CoA synthetase	7.3	74089	106938
80	ASA2	gi|159477287	Mitochondrial F1F0 ATP synthase associated 45.5 kDa protein	9.6	48383	131696
103	ASSD1	gi|159473875	Aspartate semialdehyde dehydrogenase	9.2	40138	411619
39	ATPA	gi|41179050	ATP synthase CF1 alpha subunit	5.4	54832	743857
40	ATPA	gi|41179050	ATP synthase CF1 alpha subunit	5.4	54832	283203
41	ATPA	gi|41179050	ATP synthase CF1 alpha subunit	5.4	54832	1223927
43	ATPA	gi|41179050	ATP synthase CF1 alpha subunit	5.4	54832	3810462
14	ATPvA1	gi|159480680	Vacuolar ATP synthase, subunit A	5.7	68921	132891
59	BCR1	gi|159488652	Biotin carboxylase, acetyl-CoA carboxylase component	9.0	52308	354966
60	BLD10	gi|159489304	Basal body protein	5.0	174819	1106041
61	BLD10	gi|159489304	Basal body protein	5.0	174819	315177
62	BLD10	gi|159489304	Basal body protein	5.0	174819	560223
111	CYN38	gi|159467709	Peptidyl-prolyl cis-trans isomerase, cyclophilin-type	5.4	44781	528179
79	EFTU_III	gi|41179007	Elongation factor Tu	5.9	45772	751191
100	FBA3	gi|159485250	Fructose-1,6-bisphosphate aldolase	8.9	41301	2007141
194	FBA3	gi|159485250	Fructose-1,6-bisphosphate aldolase	8.9	41301	1881225
200	FBA3	gi|159485250	Fructose-1,6-bisphosphate aldolase	8.9	41301	357707
219	FBP1	gi|159465323	Fructose-1,6-bisphosphatase	5.6	44929	203216
202	FTSH1	gi|159465357	Membrane AAA-metalloprotease	5.6	77727	119047
23	FTSH2	gi|159478022	Membrane AAA-metalloprotease	6.2	74509	376353
170	GAD1	gi|159491066	UDP-D-glucuronic acid decarboxylase	8.7	37274	259532
152	GBP1	gi|159463672	G-strand telomere binding protein 1	7.6	24160	702757
58	GCSL	gi|159474092	Dihydrolipoyl dehydrogenase	9.3	52905	175803
93	IDA5	gi|159482014	Actin	5.3	42094	251251
78	IF4A	gi|159466510	Eukaryotic initiation factor 4A-like protein	5.5	47309	136610
129	IPY1	gi|159489184	Inorganic pyrophosphatase	6.4	31342	1113052
122	LHCB5	gi|159475641	Minor chlorophyll a-b binding protein of photosystem II	5.4	30695	1712493
205	LHCBM1	gi|20269804	Major light-harvesting complex II protein m1	6.0	27605	2194002
119	MDH1	gi|159469941	Malate dehydrogenase	8.5	36864	1282008
120	MDH1	gi|159469941	Malate dehydrogenase	8.5	36864	262435
37	MMSDH	gi|159475673	Methylmalonate semi-aldehyde dehydrogenase	8.1	58580	182674
146	PDI2	gi|159462776	Protein disulfide isomerase	8.8	27447	176123
88	PGK1	gi|159482940	Phosphoglycerate kinase	8.9	49172	1979826
221	PGM1b	gi|159476226	Phosphoglycerate mutase	5.6	60921	161753
102	PRK1	gi|159471788	Phosphoribulokinase	9.0	42151	323308
216	PRK1	gi|159471788	Phosphoribulokinase	9.0	42151	173323
183	PSBO	gi|159473144	Oxygen-evolving enhancer protein 1 of photosystem II	8.3	30732	5260813
33	PYK1	gi|159469714	Pyruvate kinase	6.7	55233	216722
64	QCR1	gi|159477849	Ubiquinol:cytochrome c oxidoreductase 50 kDa core 1 subunit	5.9	55248	115110
48	RBCL	gi|41179049	Ribulose-1,5-bisphosphate carboxylase/oxygenase large subunit	6.1	53193	163996
187	RBCL	gi|41179049	Ribulose-1,5-bisphosphate carboxylase/oxygenase large subunit	6.1	53193	3590194
73	THS1	gi|159480894	Threonine synthase	9.4	54835	126623
7	TRK1	gi|159487741	Transketolase	7.1	78352	460717
55	TUA1	gi|159467393	Alpha tubulin 1	5.0	50182	283438
45	TUB2	gi|159471706	Beta tubulin 2	4.7	50157	341849
53	TUB2	gi|159471706	Beta tubulin 2	4.7	50157	514491
54	TUB2	gi|159471706	Beta tubulin 2	4.8	50157	478728
*74*	*nd*	*gi|159468534*	*Predicted protein*	*6.3*	*42690*	*575642*
*107*	*nd*	*gi|159478405*	*Hypothetical protein CHLREDRAFT_185022*	*5.5*	*36849*	*115745*
*224*	*nd*	*gi|159491024*	*Hypothetical protein*	*10.3*	*33272*	*94742*

All identified proteins were found to belong to C. reinhardtii. Identified spots are sorted by alphabetic order of corresponding Gene Name for visual convenience. The spot volume of the G-Dye100-labeled internal standard (I.S.) in the Master gel is also provided as a reference to assess protein abundance in 2D-gels. The spots which passed the initial PLSR and MLR-based screening are presented in the upper part of the table and highlighted in bold (see also Additional file 3). pI, isoelectric point; MW, molecular weight.

##### Additional assays

The cellular contents of triglycerides and of some fatty acids and pigments were also considered as continuous biological variables, as well as the respiratory and photosynthetic activities previously reported through DOE approach (Gérin et al., 2014). The respective units of these variables and the analytical methods employed to quantify them are summarized in Table 2 (for more details, read the Material and Methods Section). A complete list of the experimental values is provided in Additional file 2.

#### General features of the data sets

As shown in Additional file 2, one value of protein abundance is available for each protein spot and each culture condition of the DOE (no missing value in the protein data set). Concerning additional assays, there is one missing value for palmitic, stearic, and linolenic acids (item 42 of the DOE), two missing values for γ-linolenic acid (items 9 and 42 of the DOE) and three missing values for oleic acid (items 9, 31, and 42 of the DOE). The other additional assays have no missing values. DOE item 42, which is the most frequent missing value among the additional assays, is a center point of the DOE for which two identical measurements exist (items 4 and 41). As explained in the Material and Methods Section, all statistical analyses were performed without imputation of the missing values except hierarchical clustering.

#### Screening of the biological variables influenced by one or several environmental variables

An initial screening was carried out to highlight the protein spots and additional assays relevantly influenced by the environmental variables. This first selection was performed to point out the biological variables for which no further analysis of regulation was necessary (i.e., those for which there was no significant influence of light, carbon, or nitrogen). This screening was performed through a methodology coupling partial least squares regression (PLSR) and multiple linear regression (MLR; the reader is invited to refer to the Material and Methods Section for an extensive description of the procedure).

The screening led to the selection of 83 protein spots over 135 (61%) and 15 additional assays over 19 (79%). The results of the screening are described in Additional file 3 for protein spots and in Additional file 4 for additional assays (protein spots of interest are pointed out by orange arrows surrounded by Master numbers in Figure 2 and are presented in the upper part of Table 4 in bold characters).

In numerous cases, two or more spots in 2D-gels were identified as the same protein (91 spots corresponding to 31 different proteins, see Table 4). These observations are due to post-translational modifications generating slight modifications of the isoelectric point (e.g., phosphorylations, deamidations, oxidations) and molecular weight (e.g., complex glycosylations, differences in N- and C-terminal processing; Nield et al., 2004; Mathy and Sluse, 2008). Only two proteins with multi-identification, FBA3 (three spots) and MDH1 (two spots), did not pass the initial screening for any spot. Less than half of the spots were selected for ATPA (two over six), BLD10 (one over four), and PRK1 (one over three). For the remaining 26 proteins, there were at least 50% of the spots which passed the screening (Additional file 3).

Interestingly, the proteins that are not passing the selection procedure seem to exhibit specificities with regard to their sub-cellular localization or biological function (Table 4 and Additional file 3). The results obtained for the subunits of ATP synthase indicate that the importance of their regulation by light, carbon and nitrogen could mostly depend on their respective cellular compartments: most of the mitochondrial subunits (three over four spots including ASA1, ASA2, and ATP2) passed the screening whereas the chloroplastic and vacuolar subunits were globally rejected (this rejection concerns five spots over eight including ATPA and ATPvA1). A gene set enrichment analysis was carried out in order to verify this qualitative observation (Additional file 5). The “GO cellular component complete” annotation data set of the PANTHER database, in which genes and proteins are classified according to their sub-cellular location, was used. The analysis was carried out by comparing the ATP synthase items found in the unselected protein data set against a reference list consisting of all identified ATP synthase subunits (see Table 4). As shown in Additional file 5, cellular component groups referring to chloroplastic locations exhibited the highest fold enrichment (two folds, positive) with the lowest *p*-value (0.25 without Bonferroni correction for multiple testing). Most of the vacuolar and cell periphery classes were also characterized by a two folds positive enrichment (*p* = 0.437; both locations are known to specifically contain V-type ATPases, as opposed to the F-type ATP synthases found in chloroplasts and mitochondria). The lowest fold enrichment (more than five folds, negative) was observed for cellular component groups referring to mitochondrial locations (*p* = 0.562). These features tend to support the observations mentioned earlier, but should nevertheless be considered cautiously with regards to the lack of statistical robustness since none of the *p*-values was significant (*p* > 0.05). This is probably due to the very low number of distinct proteins used to perform the enrichment analysis (only four mapped protein IDs in the reference list and two within the list of unselected ATP synthase subunits; Additional file 5). Moreover, a feature of the technique is to treat each protein as a unique entry (i.e., a unique protein ID) without consideration of the number of spots that are found in each list.

For glycolytic enzymes and cytoskeleton and flagellar constituents, the weakness of the environmental regulation rather seems to be related to the metabolic role of the proteins, without apparent influence of their sub-cellular localization. Among the eight protein spots identified as cytoskeleton and flagellar constituents (basal body protein BLD10, tubulins α and β, actin IDA5), most appear not to be relevantly influenced by the environmental variables (only one BLD10 spot over four passed the screening). None of the glycolytic enzymes (five protein spots including FBA3, PGM1b, and PYK1) were selected through the applied procedure. These results suggest that the capacities of glycolysis as well as chloroplastic and vacuolar ATP synthesis were possibly not much influenced by the overall changes of light, carbon and inorganic nitrogen applied in the present study. This is the same for the composition of the cytoskeleton.

#### Detection and characterization of discrete groups of co-regulated biological variables

##### Partitioning of protein spots through hierarchical clustering

Hierarchical clustering was performed to partition protein spots according to the similarities of their abundance pattern among the culture conditions defined in the DOE.

**Figure 3:**
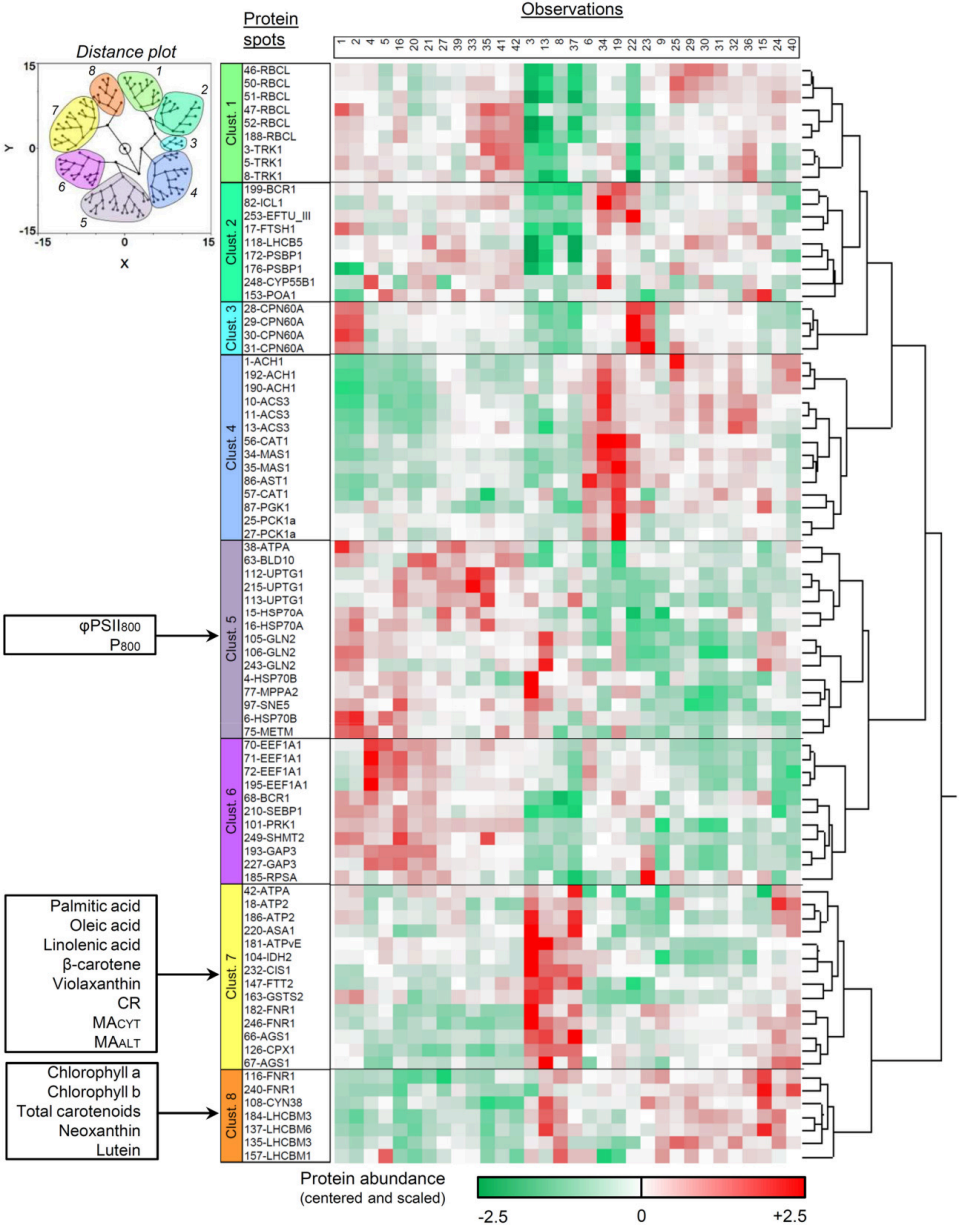
**Hierarchical clustering analysis of protein spot abundance pattern upon DOE conditions**. Only the spots which passed the initial PLSR- and MLR-based screening were included. Protein abundance is illustrated as a dendrogram with a green-to-red color scale, and the numbering of culture conditions corresponds to that in Additional file 2. A plot illustrating the 2D-distance among the spots is also provided (upper left) to facilitate cluster visualization. The allocation of the additional assays within the different protein clusters was assessed by a separate hierarchical clustering analysis integrating all biological variables. Clust., cluster.

Results are presented in Figure 3 as a dendrogram with a color range (from green to red) illustrating protein abundance in the different DOE conditions. A two-dimensional distance plot is also displayed to facilitate cluster visualization. Eight protein spot clusters can be defined according to the general abundance pattern among the tested conditions (Figure 3). For 28 proteins over 31 with multi-identifications, the different spots were clustered together (partitioning among two distinct clusters only for BCR1, ATPA, and FNR1). The protein function(s) and cellular location(s) within each cluster were searched in the Pathway Tools section of the ChlamyCyc database (May et al., 2009) and summarized in Table 5.

**Table 5:** **Protein function(s) and sub-cellular localization(s) as found in the ChlamyCyc database**.

**Gene name**	**Function(s)**	**Location(s)**
**CLUSTER 1**		
RBCL, TRK1	Calvin cycle	Chloroplast
**CLUSTER 2**		
BCR1	Fatty acid biosynthesis	Chloroplast
CYP55B1	Nitric oxide detoxification	*nd*
EFTU_III	Protein elongation	Chloroplast
FTSH1	Photosystem maintenance	Chloroplast
ICL1	Glyoxylate cycle	Mitochondrion, peroxisome
LHCB5	Light-harvesting antennae	Chloroplast
POA1	Proteasome	Cytosol
PSBP1	Photosynthetic O_2_ evolution	Chloroplast
**CLUSTER 3**		
CPN60A	Protein folding and stability	Chloroplast
**CLUSTER 4**		
ACH1	TCA cycle, glyoxylate cycle	Mitochondrion
ACS3	Acetate conversion to acetyl-CoA	Cytosol, mitochondrion
AST1	Amino-acid interconversion, anaplerosis, malate-oxaloacetate shuttle	Chloroplast, mitochondrion
CAT1	H_2_O_2_ detoxification	Mitochondrion, peroxisome
MAS1	Glyoxylate cycle	Peroxisome
PCK1a	Gluconeogenesis	Cytosol
PGK1	Glycolysis, gluconeogenesis, Calvin cycle	Chloroplast
**CLUSTER 5**		
ATPA	ATP synthase, F_1_ subunit component	chloroplast, thylakoid membrane
BLD10	Flagellum assembly and structure	Cytosol
GLN2	GS/GOGAT cycle	Chloroplast
HSP70A	Protein folding and stabilization	Cytosol
HSP70B	Photosystem assembly and maintenance	Chloroplast
METM	S-adenosylmethionine biosynthesis	Cytosol, mitochondrion
MPPA2	Protein import to mitochondria	Mitochondrion
SNE5	Cell-wall and secondary metabolite biosynthesis	*nd*
UPTG1	Protein glycosylation	Cytosol, mitochondrion
**CLUSTER 6**		
BCR1	Fatty acid biosynthesis	Chloroplast
EEF1A1, RPSA	Protein elongation	Cytosol
GAP3, PRK1, SEBP1	Calvin cycle	Chloroplast
SHMT2	Photorespiration	Mitochondrion, cytosol
**CLUSTER 7**		
AGS1	Arginine biosynthesis	Chloroplast
ASA1	ATP synthase, F_1_ subunit component	Mitochondrion
ATP2	ATP synthase, F_1_ subunit component	Mitochondrion, inner membrane
ATPA	ATP synthase, F1 subunit component	Chloroplast, thylakoid membrane
ATPvE	ATP-dependent proton pump for active transport processes	Vacuolar membrane
CIS1, IDH2	TCA cycle, glyoxylate cycle	Mitochondrion
CPX1	Chlorophyll and heme biosynthesis	Chloroplast
FNR1	Photosynthetic electron transport	Chloroplast
FTT2	Enzymatic activity regulation	Mitochondrion
GSTS2	Peroxidized lipids and proteins detoxification	*nd*
**CLUSTER 8**		
CYN38	Photosystem assembly and stabilization	Chloroplast stroma, thylakoid lumen
FNR1	Photosynthetic electron transport	Chloroplast
LHCBM1, LHCBM3, LHCBM6	Light-harvesting antennae	Chloroplast, thylakoid membrane

Proteins are denominated by their corresponding Gene Name (see Table 4 for a complete description) and classified by cluster for visual convenience.

Most proteins found in cluster 4 are involved in pathways related to acetate assimilation (e.g., acetyl-CoA synthesis, glyoxylate cycle, TCA cycle, and gluconeogenesis; Figure 3, Table 5). Acetyl-CoA is generated from acetate by acetyl-CoA synthetase and is then metabolized through the glyoxylate and TCA cycles. The reducing equivalents and C_4_ intermediates produced by these pathways can then be directed to gluconeogenesis, as previously shown in *C. reinhardtii* (Johnson and Alric, 2012). Aspartate aminotransferase (involved in anaplerosis and reductant transport) and catalase (participating to ROS detoxification) were also partitioned in the same cluster. This observation might be related to the higher electron input possibly induced by acetate assimilation. Such a feature could heighten the intracellular redox state and the ROS production rate, and make necessary to develop higher capacities of reductant transport and ROS detoxification.

In cluster 7, most proteins are related to bioenergetic processes: enzymes of the TCA cycle, components of the mitochondrial ATP synthase, coproporphyrinogen III oxidase (precursor of heme and chlorophyll), and proteins involved in cell redox signaling (glutathione-S-transferase and 14-3-3 protein FTT2; Foyer and Noctor, 2003; Roberts, 2003). Argininosuccinate synthase (which catalyzes the last, irreversible reaction of arginine biosynthesis) can also be found in this cluster. The carbon skeletons, reducing equivalents and ATP molecules generated by mitochondrial catabolism are important substrates for amino acid biosynthesis, as extensively reported (Turpin, 1991; Huppe and Turpin, 1994; Foyer et al., 2011). With this regard, the partitioning of biological variables related to mitochondrial catabolism and amino acid biosynthesis into the same cluster might reflect the need to coordinately regulate the capacity of both groups of pathways.

Most members of cluster 8 are chloroplastic proteins involved in the assembly, the architecture and/or the stabilization of core photosystems and light-harvesting antennae. Ferredoxin-NADP reductase, an enzyme participating to the photosynthetic electron transport, can also be found there (two spots in cluster 7 and two spots in cluster 8). In cluster 5, most proteins are related to protein biosynthesis, maturation, stabilization, targeting and/or assembly into complex structures. In clusters 1 and 6, proteins are all involved in anabolic processes (especially the Calvin cycle). Finally, cluster 2 can be described as a tote-bag in which there is no clear tendency with regard to the general function of proteins.

Gene set enrichment analyses of pathways were carried out to verify the co-segregation of proteins participating to common metabolic functions. The analyses were performed in the “GO Pathways” annotation data set of the PANTHER database. The proteins within each cluster were compared to a reference list made of all proteins used to perform hierarchical clustering (47 different proteins). The pathways exhibiting a positive enrichment comparatively to the reference list are displayed in Additional file 6 for each cluster. Among the 47 proteins in the reference list, 45 could be mapped to at least one pathway entry in the database. Unfortunately, for 28 of these proteins, this entry was the unclassified category. In clusters 1, 2, 3, 6, and 8, the unclassified category contained nearly all proteins and showed a positive enrichment of about 1.5-fold (except in cluster 6 for which the enrichment was only worth 1.15). In cluster 4 (three unclassified proteins over seven), acetate utilization and asparagine/aspartate biosynthesis exhibited the highest fold enrichment (6.43; Additional file 6). In cluster 5 (four unclassified proteins over seven), a five-folds enrichment was observed for two pathways related to amino acid biosynthesis: glutamine/glutamate conversion and S-adenosylmethionine biosynthesis. In cluster 7 (five unclassified proteins over ten), pathways related to bioenergetics (heme biosynthesis and pyruvate metabolism) and amino acid biosynthesis (leucine and arginine) showed the highest fold enrichment (4.50). The same result was also obtained for two redox signaling pathways as well as for the degradation of ascorbate (which is a powerful antioxidant; Hüttemann et al., 2007; Smirnoff, 2011; Lamb et al., 2015). These results collected for clusters 4, 5, and 7 are in agreement with the considerations mentioned earlier with regard to the metabolic function of proteins. It should be noticed that nearly all *p*-values of the enrichment analysis are unsignificant (*p* > 0.05; see Additional file 6); results should therefore be considered cautiously due to the lack of statistical robustness. The reasons for that could be the same as those described in Section Screening of the Biological Variables Influenced by One or Several Environmental Variables.

##### Integration of the additional assays within specific protein clusters

Hierarchical clustering was reiterated by also including additional assays in the analysis, together with protein spots. The purpose of that was to partition the assays in the different protein clusters according to pattern similarities among the DOE conditions. Results are summarized in Figure 3.

All respiratory parameters (CR, MA_CYT_, and MA_ALT_) and fatty acids (palmitic, oleic, and linolenic acids) are associated with protein cluster 7. Palmitic, oleic, and linolenic acids constitute highly-energetic substrates for β-oxidation in the mitochondrion, and are known to mediate the activity of the mitochondrial uncoupling proteins (Jezek et al., 1998). Moreover, linolenic acid is the most abundant fatty acid found in plant thylakoid membranes (Murphy, 1986). β-carotene and violaxanthin also exhibit pattern similarities with protein cluster 7. They are the only carotenoids for which the biological function cannot be substituted by other pigments in case of mutational deletion, and are thought to protect the photosynthetic apparatus from photo-oxidative damages (Trebst, 2003). The remaining pigments (chlorophylls *a* and *b*, total carotenoids, neoxanthin, lutein) were rather partitioned with protein cluster 8. For φPSII_800_ and P_800_, the dependence upon DOE conditions is related to protein cluster 5.

The term “cluster” will be used thereafter to designate each group of biological variables (proteins and assays) exhibiting a similar pattern among the DOE conditions, as shown by hierarchical clustering.

##### Characterization of the cluster-specific attributes by multivariate analysis

**Figure 4:**
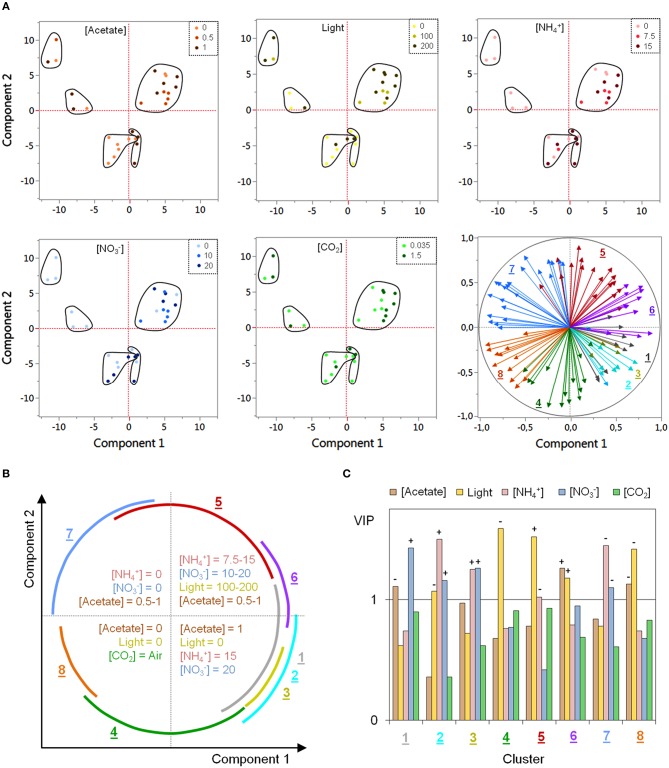
**PCA and PLSR analyses of in-cluster regulatory specificities regarding the DOE conditions**. PCA was performed with all biological variables in the same analysis whereas one PLSR was performed for each cluster. **(A)** Results of PCA. The score plot was replicated in five copies so as to enable to mark the observations according to the values taken by each environmental variable in DOE. In the loading plot (at the bottom right), vectors of the biological variables are colored according to their respective cluster. **(B)** Biplot-like scheme summarizing (i) the regulatory tendencies observed within each quadrant of the score plot regarding the DOE conditions and (ii) the angular covering by the vectors of each cluster within the loading plot. **(C)** Results of PLSRs as the variable importance in projection (VIP) of the environmental factors for each cluster. The sign of the coefficients within PLSR models is provided for VIP values exceeding 1.

A principal component analysis (PCA) was performed with all biological variables in order to characterize cluster-specific regulatory tendencies with regard to the particularities of the DOE conditions. Figure 4A shows the results of the PCA as the corresponding score plot and loading plot, based on the two first principal components. The first and the second components account for 29.7 and 24.1% of the variability, respectively, with only 9.6% for the third component (see Additional file 7).

In the loading plot (bottom-right of Figure 4A), the vectors of the biological variables are colored according to their respective cluster. As expected, biological variables within each cluster appear to be grouped together as vector bundles pointing toward a specific direction. As illustrated in Figure 4A, the correlation among biological variables is the highest within clusters 3, 6, and 8, as evidenced by the narrow angle covered by their vector bundles. In the other clusters however, the observation of a much more important angle (close to 90°, with a maximal amplitude for cluster 5) indicates that the correlation between some biological variables can be very weak despite the high correlation between neighboring vectors (the correlation matrix of each cluster is displayed in Additional file 8). For example, in cluster 5, a correlation of 0.93 (highest value within the cluster) is observed between two isoforms of UPTG1 (spots 112 and 215) but the correlation is of only –0.18 between 4-HSP70B and 3-BLD10.

In order to assign the cluster-specific grouping of biological variables to specificities of the DOE culture conditions, the score plot was reproduced in five identical copies (one per environmental variable) and each observation was marked with its respective DOE value using a specific color scale (Figure 4A). A shown there, the observations can be divided into five groups according to their relative position to the first and the second principal components. The distinction between the two groups of the inferior quadrants is essentially due to the third principal component (data not shown). Interestingly, each group of observations exhibits specific tendencies regarding the value of one or several environmental variable(s). Figure 4B summarizes the tendencies observed within each quadrant and also displays angular covering by the vectors of each cluster under the form of a biplot-like scheme.

In addition, in-cluster PLSRs were carried out as a supplementary way to assess regulatory specificities. Variable importance in projection (VIP) of each environmental variable is displayed in Figure 4C for the different clusters (see Additional file 7 for details about PLSR results). For VIPs exceeding the cutoff value of 1, the sign of the coefficient in the PLSR models is also provided.

As highlighted in Figures 4B,C, negatively correlated clusters (cluster 6 vs. 8; cluster 4 vs. 5; clusters 1-2-3 vs. 7) exhibit an opposite regulation by specific environmental variables. Biological variables found in clusters 6 and 8 are both controlled by acetate concentration and light intensity with a positive influence of these factors in cluster 6 and a negative one in cluster 8. For the members of clusters 4 and 5, there is a substantial effect of light intensity which appears to be negative in cluster 4 and positive in cluster 5. Finally, nitrate and/or ammonium concentrations seem to be the most important factors regulating biological variables in clusters 1, 2, 3, and 7, with a positive influence in clusters 1-2-3 and a negative one in cluster 7. It is worth noticing that neither PCA nor PLSR enable the visualization of more complex effects than linear ones. Results presented in Figures 4B,C are therefore likely to provide an incomplete overview of in-cluster regulatory tendencies.

Altogether, PCA and PLSR results indicate that regulatory tendencies exist within each cluster with some in-cluster subtle regulatory divergences, as suggested by the observation of a quite weak correlation among some biological variables. These divergences were further characterized by an analysis of covariance (ANCOVA) through MLR (one model per cluster), by introducing the identity of the biological variables as a categorical model predictor (for details, read the Material and Methods Section). ANCOVA results are presented in Additional file 9. All models are significant (*p* < 0.0001) with relatively high values of *R*^2^ adjusted (0.62 on average) and low fitting root mean squared errors (*RMSE*_*F*_ ≤ 33% of the response average scale). For each individual biological variable, the effects of the different environmental factors were statistically compared to the overall regulation within the cluster, and the significant differences (*p* ≤ 0.05) were further characterized by their respective β-weights (standardized regression coefficients). This approach enabled on the one hand to identify the biological variables exhibiting an outlying regulation within each cluster, and on the other hand to determine which environmental variables were responsible for this divergence (see the summary scheme in Additional file 9). Consistently with PCA results, ANCOVA demonstrates that the environmental regulation is quite homogenous in clusters 3, 6, and 8 but shows a more important proportion of discrete divergences in the other clusters.

#### Independent modeling of the dependence of each biological variable upon light, carbon, and nitrogen

The influence of the environmental factors (Table 2) was modeled independently for each biological variable through MLR. Single effects and second-order interactions between environmental variables were considered, as well as linear and quadratic effects for the continuous ones (for details, read the Material and Methods Section). Prior to MLR modeling, stepwise regression was carried out with minimum AICc (corrected Akaike information criterion) as stopping rule in order to reduce the number of coefficients and limit the probability of overfitting (Gérin et al., 2014).

##### Model parameters and cross-validation

The model parameters and the regression equation of each biological variable are provided in Additional file 10. The values predicted by this equation for the different DOE culture conditions are listed in Additional file 11. On average for all biological variables, *RMSE*_*F*_ is worth 40% of the response average scale with a standard deviation of 11%, and the mean *R*^2^ adjusted is equal to 0.61 with a standard deviation of 15%. The whole-model ANOVA *p*-values are statistically significant for every biological variable, and the lack-of-fit is significant for 101-PRK1 only (*p* = 0.0047). These results indicate that the effects included in the models are likely to be sufficient to explain most of the variability of the biological variables.

Models were cross-validated by the *k*-fold method with *k* = 4 in order to spot potential overfitting. Data subsets are described in Additional file 11. Cross-validation results are displayed in Additional file 12 as superimposed bar charts enabling comparison between the cross-validation root-mean-squared error (*RMSE*_*CV*_) and both the training *RMSE*_*F*_ and the response average scale (references to assess the extent of *RMSE*_*CV*_). On average, *RMSE*_*CV*_ is worth 51% of the response average scale with a standard deviation of 15%. *RMSE*_*CV*_ exceeds the training *RMSE*_*F*_ by 32% on average (= 12% of the response average scale) with a standard deviation of 19% (= 7% of the response average scale). Since *RMSE*_*CV*_ is not harshly above *RMSE*_*F*_, models are likely not to overfit for most biological variables. As shown in bar charts (Additional file 12), exceptions to this assumption could be 193-GAP, 108-CYN38, 182-FNR1, 29-CPN60A, and 71-EEF1A (*RMSE*_*CV*_ exceeds *RMSE*_*F*_ by more than two-thirds, suggesting that the less significant factors—with 0.01 < *p* < 0.05—should be considered cautiously).

##### Relative importance and mathematical profile of the environmental variables

**Figure 5:**
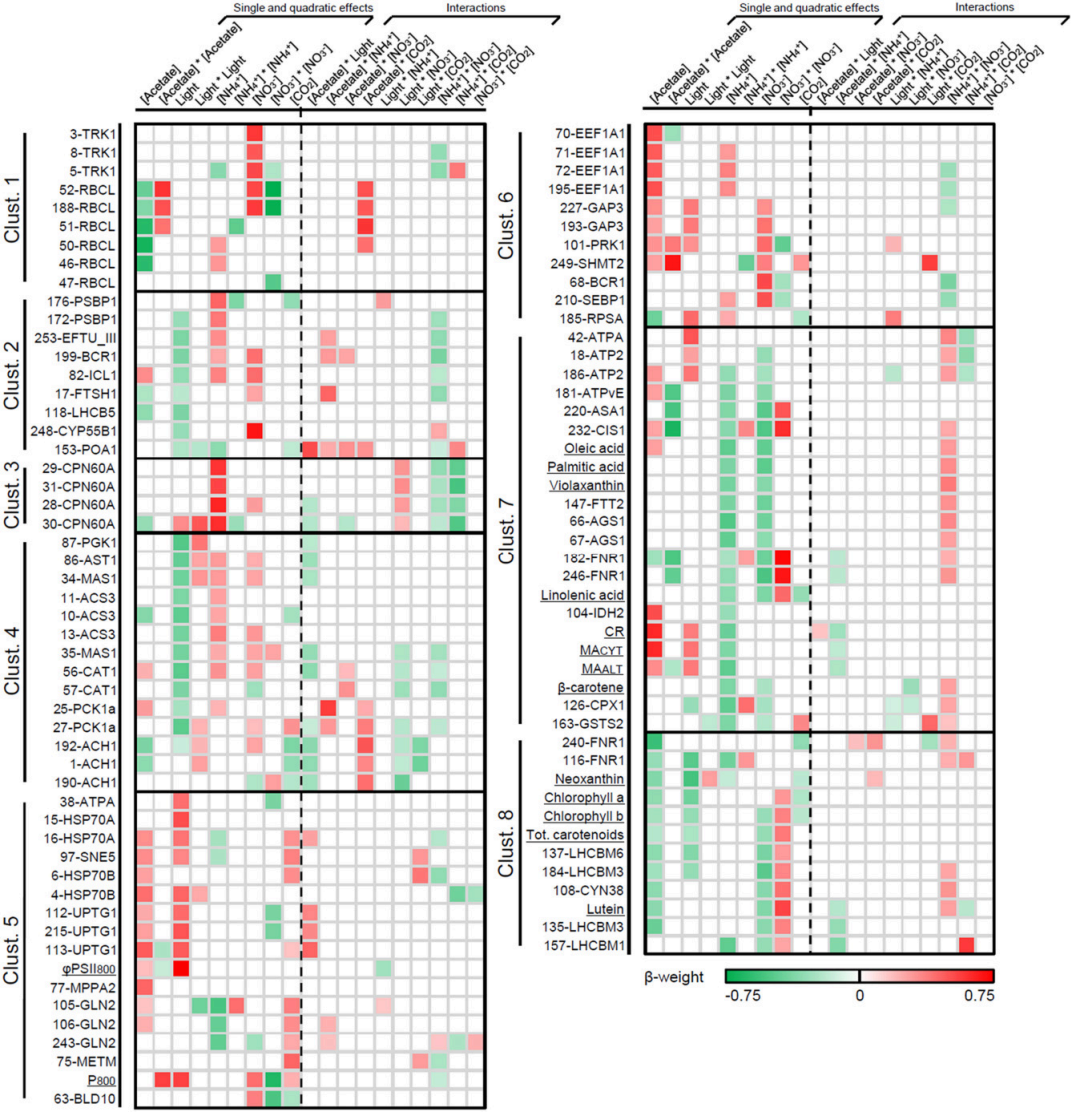
**β-weights associated with the statistically significant effects (*p* ≤ 0.05) of MLR models for individual biological variables**. β-weights are illustrated as a green-to-red color scale; empty cases are for insignificant effects or effects which were not selected by stepwise regression (see Additional File 10 for raw data). Biological variables are classified by cluster (Figure 3); within each cluster, they were sorted such as to facilitate the visual comparison of their respective regulation patterns. Clust., cluster.

Figure 5 illustrates the β-weights associated to the statistically significant coefficients (*p* ≤ 0.05) as a green-to-red heat map. Protein spots and additional assays are sorted by cluster, and the empty cells are either for insignificant or stepwise-unselected effects. A complete list of β-weights and *p*-values is provided in Additional file 10.

As illustrated in Figure 5, biological variables are mostly regulated through single, linear effects of the environmental factors (over 50% of significant linear coefficients for the continuous variables and 26% for CO_2_ concentration). Only 16% of the quadratic coefficients are globally significant but this proportion reaches 29% for nitrate concentration. The second-order interaction between nitrate and ammonium is statistically significant for 48% of the biological variables, with only 2–16% for the other interactions. This observation suggests that the nature of the inorganic nitrogen source and the balance between its different molecular species are likely to be key regulators of cellular metabolism.

**Figure 6:**
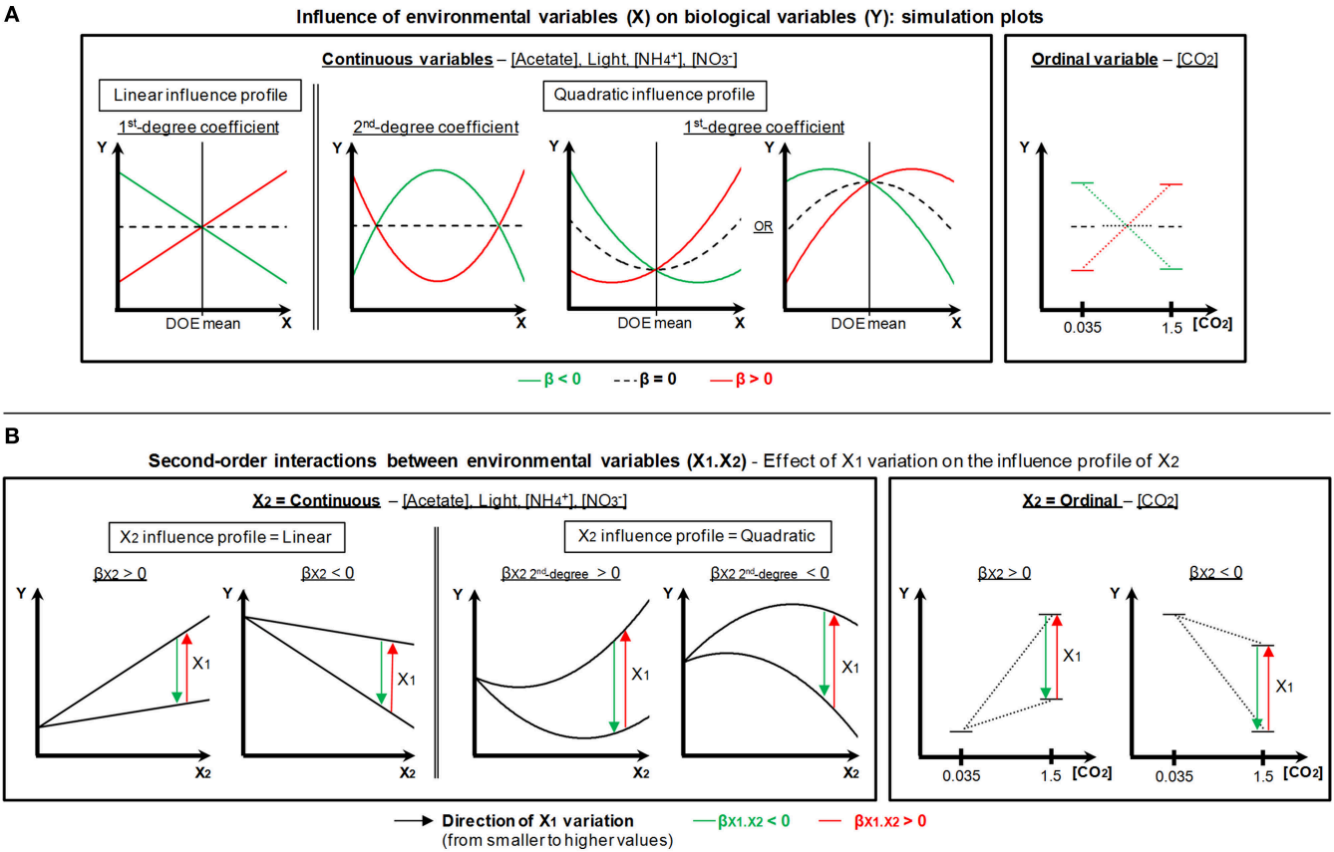
**Generalized simulation plots for MLR individual modeling of the biological variables**. This figure is the key for reading the regulation results summarized in Figure 5. **(A)** Influence profile of the environmental variables according to the type of effect (ordinal, continuous linear or continuous quadratic) in relationship with the sign and magnitude of the associated β-weight(s). **(B)** Second-order interactions between environmental variables (X_1_ and X_2_) and simulates the incidence of X_1_ variation on the influence profile of X_2_ in relationship with the value of the β-weight of the interaction. Possible variations of X_2_ graph intercept as a function of X_1_ are not represented on the schemes.

Nearly all biological variables are nonetheless regulated through complex superimpositions of linear effects, quadratic effects and/or second-order interactions of the environmental variables (Figure 5). That renders the visualization of regulation quite difficult and makes necessary to perform a case-by-case mathematical simulation for every biological variable and environmental factor. For technical reasons, it is not possible to present such numerous simulations here. Consequently we rather chose to build generalized simulation plots considering every possible situation (Figure 6) as a key to read the results presented in Figure 5.

In good agreement with PCA and ANCOVA, the identity, sign and relative importance of the significant coefficients are especially homogenous among biological variables in clusters 3 and 8 (Figure 5). As expected, the regulatory differences among the clusters appear to be far more remarkable than within each individual one (existence of a clear regulatory pattern unique to every cluster). The results obtained by PCA and PLSR with regard to the in-cluster regulatory specificities (Figures 4B,C) are consistent with MLR results (Figure 5).

### Description of the environmental regulation of the biological variables according to their metabolic function

#### Biological variables related to photosynthesis and protein metabolism

Most components of light-harvesting antennae (LHC proteins and pigments, cluster 8) are controlled by light intensity and acetate concentration through negative linear effects (Figure 5, Table 5). Consistently, acclimation to increasing irradiance has long been known to involve a substantial down-regulation of LHC proteins and to lower the cellular pigment amount (Neale and Melis, 1986; Falkowski and LaRoche, 1991; Teramoto et al., 2002; Durnford et al., 2003; Nield et al., 2004). Moreover, the contents of chlorophyll *a* and *b* have been reported to be decreased in *C. reinhardtii* cells grown in the presence of acetate (Boyle and Morgan, 2009). On the contrary, most of the actors of the enzymatic machinery involved in protein synthesis and maturation (clusters 5 and 6) are regulated by light intensity and acetate concentration through positive linear effects (with the exception of EEF1A for which there is no influence of light, see Figure 5). Accordingly, the cytosolic heat-shock protein HSP70A has already been reported to be induced by light at the mRNA abundance level (Von Gromoff et al., 1989).

In addition, most components of the light-harvesting antennae (cluster 8) tend to be regulated by nitrate concentration through a quadratic convex profile (minimum estimated around 12.5 mM; Figures 5, 6, Additional file 10). Interestingly, a reciprocal concave control is exerted by this factor on a series of other photosynthesis-related biological variables: P_800_ (the maximal gross O_2_ evolution) and two enzymes catalyzing regulatory steps of the Calvin cycle (sedoheptulose-1,7-bisphosphatase and phosphoribulokinase; Hahn et al., 1998; Raines, 2003). In some circumstances, P_800_ can be regarded as an indicator of the capacity of the metabolic pathways consuming the photo-generated NADPH and ATP, such as the Calvin cycle (Badger et al., 2000).

Remarkably, the enzymes involved in protein synthesis and maturation (cluster 5) are the only group of biological variables exhibiting a clear regulatory tendency upon CO_2_ concentration (positive effect; Figure 5, Table 5).

#### Biological variables related to the calvin cycle

The regulation of the Calvin cycle enzymes (clusters 1 and 6) by light, carbon and nitrogen appears to be quite heterogeneous (Figure 5, Table 5). Transketolase is mostly regulated through linear effects of nitrate concentration. Rubisco large subunit, sedoheptulose-1,7-bisphosphatase and phosphoribulokinase are controlled by quadratic effects of nitrate concentration with an additional quadratic influence of acetate concentration for RubisCO large subunit and phosphoribulokinase. A linear effect of light intensity is also observed for the latter enzyme. The abundance of glyceraldehyde-3-phosphate dehydrogenase mostly depends on linear effects of acetate concentration, nitrate concentration and light intensity. No significant effect of CO_2_ concentration could be detected for any of these enzymes, except a second-order interaction between acetate and CO_2_ concentrations for some spots of RubisCO large subunit (Figure 5). Accordingly, CO_2_ concentration has already been reported to exert no relevant influence on the cellular abundance of the latter protein (Borkhsenious et al., 1998; Mitchell et al., 2014).

#### Biological variables related to acetate assimilation

Nearly all proteins involved in acetate assimilation (cluster 3) are controlled by light intensity through negative linear effects. This concerns acetyl-CoA synthetase as well as enzymes of the glyoxylate cycle, TCA cycle and gluconeogenesis (Figure 5, Table 5). In line with these observations, light has been shown to act as a negative regulator of the glyoxylate cycle in plants (Allen et al., 1988), and lower mRNA levels have been reported for isocitrate lyase consequently to light exposure in *C. reinhardtii* (Petridou et al., 1997).

Interestingly, acetate concentration alone does not appear to significantly influence the machinery responsible for its own assimilation at the protein abundance level (Figure 5). This is consistent with the observation that the genetic expression of malate synthase and isocitrate lyase occurs in both the presence and the absence of acetate in plants (Graham et al., 1994). A negative interaction between acetate concentration and light intensity could nonetheless be detected for most proteins involved in acetate assimilation. Therefore, increasing acetate availability is expected to strengthen the negative influence exerted by light intensity on the acetate assimilatory machinery, so that the most important effect of light will be observed in case of high acetate availability (Figures 5, 6). A positive second-order interaction between acetate and CO_2_ concentrations was also detected for a few proteins participating to acetate assimilation (phosphoenolpyruvate carboxykinase and aconitase; Figure 5). This possibly indicates that the total carbon availability could contribute to control acetate assimilation to some extent.

Acetate assimilatory enzymes also tend to be regulated by nitrate and ammonium concentrations through positive linear effects (less well-defined tendency in comparison to light; Figure 5). This suggests that the abundance of these proteins could be controlled by the total availability of inorganic nitrogen. Accordingly, the mRNA levels of aconitase and phosphoenolpyruvate carboxykinase have been reported to be very sensitive to nitrogen deprivation in *C. reinhardtii* (Miller et al., 2010).

#### Biological variables related to bioenergetic processes

Nearly all the biological variables involved in bioenergetic processes found in cluster 7 are regulated by nitrate and ammonium concentrations through negative linear effects (Figure 5, Table 5). Such as for acetate assimilatory enzymes, this could indicate that the mitochondrial and chloroplastic bioenergetic pathways are very sensitive to the total availability of inorganic nitrogen at the protein abundance level. This assumption is strengthened by the observation of a positive second-order interaction between nitrate and ammonium concentrations (Figure 5). Such an interaction could contribute to improve the tightness of the metabolic response, by attenuating the negative influence of each factor when the availability of the other nitrogen source increases in the medium.

#### GroEL-homolog chaperonin CPN60A

The abundance of this protein (the only one in cluster 3) is strongly regulated by ammonium concentration through positive linear effects (Figure 5). This observation might be related a possible role of CPN60A in the enhancement of the stability of the enzymatic machinery for photosynthesis and nitrate reduction, as reported in cyanobacteria in case of thermal stress (Rajaram and Apte, 2008). The observation of a negative second-order interaction with CO_2_ concentration also indicates that the influence of ammonium might be attenuated in high CO_2_-grown cells. This effect of CO_2_ could be related to the well-known participation of CPN60A to the assembly of RubisCO holoenzyme in plants (see Hauser et al., 2015 for review).

## Discussion

The present work is focused on studying the influence of simultaneous variations of light, carbon and inorganic nitrogen on the cellular proteome of *C. reinhardtii*. For this purpose, design of experiments (DOE) and sequential multivariate analyses were used to model protein regulation upon overall environmental changes. Proteomic results were completed by additional assays for respiration, photosynthesis, and cellular contents of some lipids and pigments, and the data of these assays were integrated into proteomic results through multivariate statistics. To date, most reported efforts have been focused on studying the effects of one or two environmental variables on photosynthetic metabolism (keeping the other variables constant). Moreover, little information was available in literature concerning the mathematical influence profile of each variable and its relative weight.

Over the last decade, a very wide panel of omics-based approaches has been developed to gain deeper understanding of many aspects of cellular biology. With regard to the huge amount of data generated by these techniques, efficient bioinformatics methods of meta-analyses have been developed to reconstitute biological systems. In this context, dealing with data heterogeneity is the key problem (Fukushima et al., 2009; Mochida and Shinozaki, 2011). The use of multivariate statistical approaches could help solving this problem to some extent, by making possible to perform an overall regulation study with a single experimental design.

### The present set of sequential multivariate analyses is suitable for the characterization of the environmental regulation of *C. reinhardtii* metabolism

As already described in details, the results of the regression-based initial screening are homogenous for the different spots of proteins with multi-identifications (Additional file 3). In the individual MLR models obtained for the selected biological variables, an important proportion of the variability can be explained by light, carbon and nitrogen (Figure 5, Additional file 10). These elements indicate that the screening procedure that we used here is reliable for the present data set.

Hierarchical clustering is a key element of the present work that enabled to partition biological variables according to their regulatory similarities. Such a methodology had already been employed by Höhner et al. for the analysis of *C. reinhardtii* proteomic data to study the response to environmental changes (iron availability and trophic status; Höhner et al., 2013). The authors demonstrated that the proteins participating to a common biological function tended to be grouped together. Similarly here, hierarchical clustering enabled to partition biological variables into eight co-regulated clusters corresponding to specific biological processes: Calvin cycle (cluster 1), acetate assimilation (cluster 4), protein synthesis and maturation (cluster 5), anabolic pathways (cluster 6), processes of energy transduction (cluster 7), and composition of the photosynthetic apparatus (cluster 8; Figure 3, Table 5). The observation of such a weak number of clusters is outstanding with regard to the diversity of the environmental perturbations applied here.

An overview of the regulation by light, carbon and nitrogen within each cluster was further characterized by PCA, PLSR, and ANCOVA (Figure 4, Additional file 9). These analyses indicated the existence of slight in-cluster differences with regard to the influence of the environmental variables. These observations were particularly marked for clusters 1, 2, 4, 5, and 7, and suggested that subtle regulatory divergences could exist within each cluster despite the existence of a common pattern. These divergences among biological variables were therefore assessed by modeling the influence of light, carbon and inorganic nitrogen through MLR, independently for each protein spot and additional assay. In contrast with PCA and PLSR, these analyses enabled to simulate the mathematical influence profile of each environmental variable by taking into account quadratic effects and second-order interactions (Figures 5, 6). As expected, the differences were much less marked within the clusters than among them, confirming the existence of a clear regulatory pattern unique to every cluster.

### The present analyses provide deeper insight into the metabolic adaptations set up in response to overall environmental changes

#### Light, carbon, and inorganic nitrogen exert no influence on a series of biological variables associated to specific sub-cellular compartments or biological functions

According to the results of the initial regression-based screening, most proteins which are not substantially influenced by light, carbon or nitrogen (Table 4, Additional file 3) seem to belong to discrete sub-cellular compartments or functional groups. On the one hand, as verified by gene set enrichment analysis, this absence of environmental regulation concerns the chloroplastic and vacuolar subunits of ATP synthase. On the other hand, no incidence of light, carbon and nitrogen could be noticed for the glycolytic enzymes nor for the cytoskeleton and flagellar components analyzed here, independently of their sub-cellular localization. Previous studies indicated that light might influence glycolysis by inhibiting pyruvate kinase in *C. reinhardtii* (Xue et al., 1996). As suggested here, this possible light-mediated inhibition of glycolytic activity might not be associated to a significant decrease of the capacity of the pathway. In *C. reinhardtii*, some subunits of the chloroplastic and vacuolar ATP synthases are also known to be regulated by light through the thioredoxin system (Lemaire et al., 2004). This variation of activity does not seem to correlate with a significant modification of protein abundance.

Among the functional assays for respiration and photosynthesis, only NPQ_800_ did not pass the initial screening (Additional file 4). This may be related to the lower ability of *C. reinhardtii* to set up non-photochemical quenching of chlorophyll fluorescence in comparison with plants (Finazzi et al., 2006).

#### Influence of light, carbon, and inorganic nitrogen on the cellular metabolism

As shown in Figure 5, the regulation of most selected biological variables occurs through linear effects of light, acetate, nitrate and ammonium. For CO_2_ concentration, the number of significant coefficients (*p* ≤ 0.05) is twice lower in comparison with the other variables. Moreover, no cluster-specific regulatory tendency can be distinguished regarding this factor, except in cluster 5 in which there is a positive influence of CO_2_ for many biological variables. Remarkably, no influence of CO_2_ concentration could be detected here for Calvin cycle enzymes, including RubisCO as already reported at the abundance level (Borkhsenious et al., 1998; Mitchell et al., 2014).

We hypothesize that the weakness of CO_2_ influence could arise from two particularities of the experimental design. Firstly, the cellular density in algal cultures was relatively weak at the time of harvest (biomass: 250 μg.mL^−1^). The uptake of CO_2_ by algal cells was therefore probably not limited by the rate of CO_2_ diffusion in the aqueous phase. In these conditions, the induction of the carbon-concentrating mechanism (CCM) under 350 ppm CO_2_ might have been sufficient to buffer the variations of CO_2_ levels in the local environment of RubisCO (Moroney et al., 2011; Wang et al., 2011; Kupriyanova et al., 2013). Accordingly, the acclimation of *C. reinhardtii* cells to low CO_2_ has been associated with increased levels of several CCM proteins without modification of the abundance of RubisCO large and small subunits (Mitchell et al., 2014). Secondly, the maximal light intensity used here (200 μmol_photons_.m^−2^.s^−1^) is not high enough to induce saturation the photosynthetic electron transport chain (Sueltemeyer et al., 1986; White and Critchley, 1999). The production rates of NADPH and ATP (rather than the availability of CO_2_) are therefore likely to constitute limiting factors for the Calvin cycle in the present conditions. Altogether, these different elements might rationalize that huge modifications of CO_2_ availability (from 350 ppm to 1.5%) are shown here to induce only slight metabolic adaptations.

**Figure 7:**
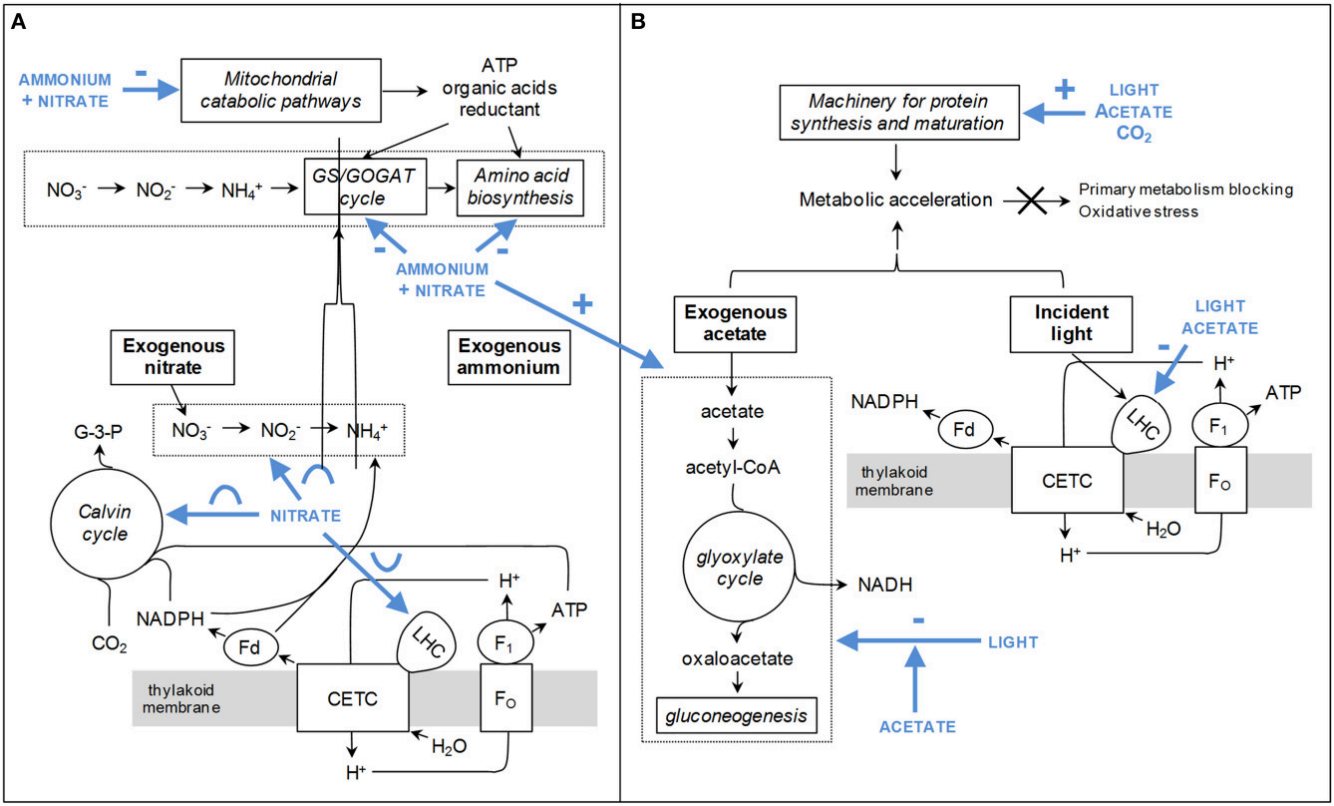
**Metabolic adaptations induced in response to variations of light, carbon, and nitrogen in the medium**. These schemes represent interpretations deduced from our results, mostly related to changes in protein abundance. **(A)** Influence of nitrate and ammonium concentrations. **(B)** Influence of light intensity and carbon availability (acetate and CO_2_). The postulated effects of the environmental factors are colored in blue and pointed out by bold arrows surrounded by specific symbols describing the type of influence: + and – are for linear profiles whereas concave and convex shapes are for quadratic profiles. CETC, chloroplastic electron transport chain; Fd, ferredoxin; G-3-P, glyceraldehyde-3-phosphate; LHC, light-harvesting complex.

The features discussed below regarding the influence of light, carbon, and inorganic nitrogen on the cellular metabolism are illustrated in Figure 7. Figure 7A is for nitrate and ammonium; Figure 7B is for light, acetate and CO_2_. These schemes represent interpretations of our results, mostly related to changes in protein abundance.

##### Influence of nitrate and ammonium

As shown in Figure 5, nitrate and ammonium exert a significant influence on biological variables related to carbon metabolism (Calvin cycle, glyoxylate cycle, gluconeogenesis) and processes of energy transduction (respiration, photosynthesis, light harvesting; see also Table 5). The predominant regulatory nitrogen form and its mathematical influence profile are specific for each pathway: for example, light-harvesting antennae components (cluster 8) are regulated by nitrate concentration through a quadratic influence profile, whereas processes of energy transduction (cluster 7) rather depend on the total availability of inorganic nitrogen through negative effects. To date, the influence of nitrate and ammonium concentrations had poorly been investigated, but dramatic effects of nitrogen deprivation on many aspects of biological functions had nonetheless been reported (Plumley and Schmidt, 1989; Turpin, 1991). Altogether, these data and the present results emphasize that the inorganic nitrogen source is a key factor controlling the energetic balance of the cell. Interestingly, for nearly half of the biological variables, a significant interaction could be detected between nitrate and ammonium concentrations (Figure 5). This suggests that the balance between these two forms also exerts a particularly important control on biological processes, probably because of the higher energetic requirements of nitrate assimilation (Fernandez et al., 2004).

*Nitrate quadratically influences the machineries for light-harvesting, photosynthesis and CO_2_ fixation: A way to adjust the photo-production of reductant, ATP and carbon skeletons to the assimilation of this N source?* The components of light-harvesting antennae (LHC proteins ant pigments in cluster 8) are shown here to be regulated by nitrate concentration through a convex profile with an inflecion point around 12.5 mM (Figures 5, 6, Additional file 10). Interestingly, several other biological variables related to photosynthesis are controlled by nitrate through a reciprocal concave profile: Calvin cycle enzymes (sedoheptulose-1,7-bisphosphatase, phosphoribulokinase, some spots of RubisCO large subunit), linolenic acid (the most abundant fatty acid in thylakoid membranes), P_800_ (the gross photosynthetic O_2_ evolution), and some spots of ferredoxin-NADP reductase (Figures 5, 6). These data suggest that certain aspects of light harvesting, photosynthetic electron transport and CO_2_ fixation are coordinately regulated by nitrate concentration. Remarkably, the observation of quadratic profiles indicates the existence of an optimal concentration of this factor for photosynthesis. The experimental elements are nevertheless insufficient to rationalize the occurrence of two types of reciprocal quadratic effects.

Photosynthesis is an important source of reductant, ATP and carbon skeletons for nitrogen assimilation (Turpin, 1991). Consequently, it can be argued that the regulation of photosynthesis by nitrate concentration arises from the need to adjust the rate of reductant, ATP and carbon skeleton production to the rate of nitrate assimilation. That had already been suggested for P_800_ in a previous publication (Gérin et al., 2014). The data reported here indicate that nitrate-induced adaptations occur at two levels: (i) at the level of the photosynthetic electron transport chain as a way to control the production of reductant and ATP, and (ii) at the level of the Calvin cycle as a way to adjust the rate of carbon skeleton production (Figure 7A). Such adaptations are likely to contribute to the regulation of the carbon-to-nitrogen balance of the cell under changing nitrate availability in the culture medium.

*Nitrogen down-regulates pathways contributing to its assimilation at the protein abundance level* A recent study reported the proteomic adaptations of algal cells upon changes of ammonium availability in the culture medium (testing of four different concentrations; Lee et al., 2012). A drastic increase of the abundance of several TCA cycle enzymes (citrate synthase, isocitrate dehydrogenase, α-ketoglutarate dehydrogenase, succinate dehydrogenase, malate dehydrogenase) and of glutamine synthetase could be detected while decreasing ammonium concentration. These results were attributed to the need to heighten the capacity for amino acid biosynthesis through the GS/GOGAT cycle and anabolic pathways (requiring organic acids as carbon skeletons) in case of low nitrogen supply (Lee et al., 2012). Here the observation that total nitrogen availability (nitrate + ammonium) exerts a negative influence on biological variables involved in mitochondrial catabolism (notably citrate synthase and isocitrate dehydrogenase) and amino acid biosynthesis (argininosuccinate synthase; see cluster 7 in Figure 5) is in agreement with this assumption. In line with that previous study, a strong negative influence of ammonium concentration on the abundance of glutamine synthetase could also be detected here (Figure 5). The present work further demonstrates that nitrogen influence prevails over the effects of light and carbon for the regulation of TCA cycle and amino acid biosynthetic enzymes at the protein abundance level (no clear regulatory tendency upon changes related to light, CO_2_ and acetate, see Figures 5, 7A).

##### Influence of light, acetate and CO_2_

*Light-mediated activation of the calvin cycle does not always correlate to higher protein abundance* Calvin cycle enzymes are known to be activated by light through redox mechanisms mediated by the thioredoxin system. That enables to accelerate the turnover of NADPH and ATP when light intensity increases, with a concomitant improvement of CO_2_ fixation (Perchorowicz et al., 1981; Brooks et al., 1988). Remarkably, the data presented here indicate that the thioredoxin activation of Calvin cycle enzymes is not always associated to higher protein abundance levels. Statistically significant coefficients were indeed detected for some enzymes (glyceraldehyde-3-phosphate dehydrogenase, phosphoribulokinase) but in other cases light was not shown to be a regulatory factor (RubisCO large subunit, sedoheptulose-1,7-bisphosphatase, transketolase; Figure 5). Accordingly, no major changes of the abundance of RubisCO large and small subunits could be detected during the dark-to-light transition in *C. reinhardtii* (Mitchell et al., 2014). Light had previously been reported to considerably enhance the mRNA levels for sedoheptulose-1,7-bisphosphatase in *C. reinhardtii* (Hahn et al., 1998) but our results indicate that this increase in transcript abundance does not result in higher protein amount.

*Adaptation to increasing irradiance heightens the capacity to assembly and protect photosystem ii reaction centers* The quantum yield of photosystem II under saturating light (φPSII_800_) was partitioned in the same cluster (n°5) as the components of the machinery for protein synthesis and maturation (Figure 3, Table 5). In this group, biological variables are positively influenced by light, acetate and CO_2_ (Figure 5). Interestingly, increasing light irradiance is known to accelerate the turnover of the D1 protein of photosystem II as a way to replace photo-damaged reaction centers (Schuster et al., 1988). In this context, the chloroplastic heat-shock protein 70B has been suggested to participate to both the protection and repair of the reaction centers (Schroda et al., 1999). Here the observation that φPSII_800_ and HSP70B are found in the same light-dependent cluster is in agreement with this postulated role of HSP70B.

φPSII_800_ and P_800_ were partitioned in the same cluster, but nonetheless differ from each other regarding the effects of acetate, nitrate and CO_2_ concentrations (Figure 5). These features might be attributable to the fact that P_800_ does not only depend on intrinsic properties of the photosynthetic apparatus, but is also modulated by interactions of photosynthesis with other metabolic pathways (Calvin cycle, photorespiration, Mehler reaction, etc.; Badger et al., 2000). The molecular mechanisms underlying P_800_ environmental regulation are therefore likely to be more complex than φPSII_800_.

*Heightening the metabolic rate and decreasing the capacities for light and acetate assimilation: a double strategy to limit the harmful effects of excess energy input?* In *C. reinhardtii*, the metabolic rate is known to be stimulated by light, acetate and CO_2_ (Sager and Granick, 1953; Yang and Gao, 2003; Boyle and Morgan, 2009). Here data demonstrate that these environmental variables exert a positive influence on the enzymatic machinery for protein synthesis and maturation (Figure 5). That could indicate that the capacity for protein turnover is increased in response to light, acetate and CO_2_, possibly as a way to support the higher metabolic rates induced by heightening these variables (Figure 7B).

Conversely, light was shown here to exert a negative influence on some pathways related to carbon assimilation, i.e., acetate metabolism (acetyl-CoA synthetase, glyoxylate cycle, TCA cycle, gluconeogenesis) and light harvesting (indirectly connected to CO_2_ fixation through the photo-production of reductant and ATP as substrates of the Calvin cycle; Figure 5). In addition, a negative influence of acetate concentration could also be detected for light-harvesting antennae components. For acetate assimilatory enzymes, the influence of this factor occurs indirectly through a negative second-order interaction with light intensity (Figure 5). This interaction strengthens the negative influence of light while increasing acetate availability, in such a way that the most important effect of light is observed in case of high acetate concentration (see also Figure 6). Accordingly, cross-talk between light and acetate signaling pathways has already been reported to play a key role in the regulation of malate synthase, a specific enzyme of the glyoxylate cycle (Nogales et al., 2004). However, varying acetate concentration alone (i.e., without changing light) appears to be insufficient to induce metabolic adaptations of the acetate assimilatory pathways (Figure 5). This observation that acetate does not exert a direct control on its own assimilation at the protein abundance level is quite remarkable.

The negative influence of light on the capacity of the photosynthetic antennae has long been known to avoid over-reducing the photosynthetic apparatus while increasing irradiance. This adaptation enables to control light energy capture and to prevent the occurrence of oxidative stress within the cell (Falkowski and LaRoche, 1991; Teramoto et al., 2002). By extension, the aforementioned adaptations related to acetate assimilation and light harvesting (Figure 5) could be a way to limit the energy input while increasing the availability of electron sources such as light and acetate. Overall, accelerating the metabolic rate and decreasing the capacities for light and acetate assimilation might be a double strategy enabling to prevent primary metabolism blocking and to limit oxidative damages consequently to increased availabilities of light and acetate (Figure 7B).

## Conclusions

Altogether, the present results support that the environmental regulation of the primary metabolism is a multifactorial issue, since nearly all biological variables were found to be influenced by complex superimpositions of linear effects, quadratic effects and/or second-order interactions of the environmental variables. That supports the usefulness of studying regulation in a context where light, carbon and nitrogen are varied simultaneously in the medium, in order to guarantee that the observations are not specific of a particular physiological state. The quadratic effects exerted by nitrate concentration on some components of the machineries for photosynthesis and CO_2_ fixation appear to us as particularly interesting. In our opinion, this influence of nitrate would deserve to be further investigated with regard to its possible consequences on primary productivity and industrial biomass yields (potential existence of an optimal nitrate concentration). If combined to omics methods exhibiting higher output levels than 2D-DIGE (gel-free proteomics, microarray, etc.), we think that the present statistical methodology could enable to considerably improve current understanding of systems biology in diverse organisms. In this context, extensive sequential statistical analyses could help dealing with heterogeneous experimental and analytical procedures to unveil hidden information in increasingly large biological data sets.

## Author contributions

GM is the author of the original idea of the work. SG and GM conceived the DOE. SG performed algal cultures, proteomic experiments as well as triglyceride and Lichtenthaler's pigment determinations with helpful advice from GM, PL, and FS for 2D-DIGE and from FF for spectroscopy. SG and GM carried out chromatographic experiments. SG performed statistical analyses with GM's contribution and wrote the manuscript. All authors read and approved the final manuscript.

## Funding

This work was supported by a “Fonds de la Recherche Fondamentale et Collective” grant (FRFC 2.4597.11) and a “Fonds de la Recherche Scientifique Médicale” grant (FRSM 3.4559.11) from the Belgian “Fonds de la Recherche Scientifique-Fonds National de la Recherche Scientifique” institution (F.R.S.-FNRS).

### Conflict of interest statement

The authors declare that the research was conducted in the absence of any commercial or financial relationships that could be construed as a potential conflict of interest.

## Abbreviations

2D-DIGE: Two dimensional-differential in-gel electrophoresis
AICc: Corrected Akaike information criterion
ANCOVA: Analysis of covariance
CCM: Carbon concentrating mechanism
DOE: Design of experiments
FAMES: Fatty acid methyl esters
IEF: Isoelectrofocalisation
I.S.: Internal standard
LHC: Light-harvesting complex
MLR: Multiple linear regression
mW: Molecular weight
PCA: Principal component analysis
pI: Isoelectric point
PLSR: Partial least squares regression
PRESS: Prediction error sum of squares
RMSE_F_: Fitting root-mean-squared error
RMSE_CV_: Cross-validation root-mean-squared error
TCA: Tricarboxylic acid
VIP: Variable importance in projection.
