# Supplementary material for: New Features on the Environmental Regulation of Metabolism Revealed by Modeling the Cellular Proteomic Adaptations Induced by Light, Carbon, and Inorganic Nitrogen in Chlamydomonas reinhardtii
Source: Front Plant Sci. 2016 Aug 9;7:1158. doi: 10.3389/fpls.2016.01158 (PMC4977305; doi:10.3389/fpls.2016.01158)
Supplement: Supplementary file 1 [file DataSheet1.DOCX]

| **2D-electrophoresis** | **G-Dye200**  (0.2 nmol) | | **G-Dye300**  (0.2 nmol) | | **G-Dye100**  (0.2 nmol) | |
| --- | --- | --- | --- | --- | --- | --- |
| Gel 1 | 25 µg of… | Sample 1 | 25 µg of… | Sample 2 | 25 µg of… | I.S. |
| Gel 2 |  | Sample 3 |  | Sample 4 |  | I.S. |
| Gel 3 |  | Sample 5 |  | Sample 6 |  | I.S. |
| Gel 4 |  | Sample 8 |  | Sample 9 |  | I.S. |
| Gel 5 |  | Sample 13 |  | Sample 15 |  | I.S. |
| Gel 6 |  | Sample 16 |  | Sample 19 |  | I.S. |
| Gel 7 |  | Sample 20 |  | Sample 21 |  | I.S. |
| Gel 8 |  | Sample 22 |  | Sample 23 |  | I.S. |
| Gel 9 |  | Sample 24 |  | Sample 25 |  | I.S. |
| Gel 10 |  | Sample 27 |  | Sample 29 |  | I.S. |
| Gel 11 |  | Sample 30 |  | Sample 31 |  | I.S. |
| Gel 12 |  | Sample 32 |  | Sample 33 |  | I.S. |
| Gel 13 |  | Sample 34 |  | Sample 35 |  | I.S. |
| Gel 14 |  | Sample 36 |  | Sample 37 |  | I.S. |
| Gel 15 |  | Sample 39 |  | Sample 40 |  | I.S. |
| Gel 16 |  | Sample 41 |  | Sample 42 |  | I.S. |

**Additional file 1.docx Sample distribution in 2D-electrophoreses**
